# Metabolomic Signatures of Biotrauma Associated with Mortality in ICU Patients Requiring Invasive Mechanical Ventilation and ECMO

**DOI:** 10.3390/metabo16070516

**Published:** 2026-07-22

**Authors:** Tiago A. H. Fonseca, Cristiana P. Von Rekowski, Rúben Araújo, Gonçalo C. Justino, M. Conceição Oliveira, Luís Bento, Cecília R. C. Calado

**Affiliations:** 1 NMS—NOVA Medical School, FCM—Faculdade de Ciências Médicas, Universidade NOVA de Lisboa, Campo dos Mártires da Pátria 130, 1169-056 Lisbon, Portugal; tiago.alexandre.hf@gmail.com (T.A.H.F.); rubenalexandredinisaraujo@gmail.com (R.A.); 2 ISEL—Instituto Superior de Engenharia de Lisboa, Instituto Politécnico de Lisboa, Rua Conselheiro Emídio Navarro 1, 1959-007 Lisbon, Portugal; 3 CHRC—Comprehensive Health Research Centre, Universidade NOVA de Lisboa, 1150-082 Lisbon, Portugal; 4 Centro de Química Estrutural—Institute of Molecular Sciences, Instituto Superior Técnico, Universidade de Lisboa, Av. Rovisco Pais 1, 1049-001 Lisbon, Portugal; goncalo.justino@tecnico.ulisboa.pt (G.C.J.); conceicao.oliveira@tecnico.ulisboa.pt (M.C.O.); 5 CHRC—Comprehensive Health Research Centre, NMS—NOVA Medical School, FCM—Faculdade de Ciências Médicas, Universidade NOVA de Lisboa, 1169-056 Lisbon, Portugal; luis.bento@ulssjose.min-saude.pt; 6 Intensive Care Department, ULS São José—Unidade Local de Saúde São José, Rua José António Serrano, 1150-199 Lisbon, Portugal; 7 CCAL—Centro Clínico Académico de Lisboa, 1649-028 Lisbon, Portugal; 8 iBB—Institute for Bioengineering and Biosciences, i4HB—The Associate Laboratory Institute for Health and Bioeconomy, IST—Instituto Superior Técnico, Universidade de Lisboa, Av. Rovisco Pais, 1049-001 Lisbon, Portugal

**Keywords:** ICU mortality prediction, fourier-transform infrared spectroscopy (FTIRS), UHPLC-HRMS, metabolomics, proteomics, multivariate logistic regression, machine learning

## Abstract

**Background**: Biotrauma from invasive mechanical ventilation (IMV) and extracorporeal membrane oxygenation (ECMO) drives systemic inflammation, metabolic dysregulation, and organ dysfunction in critically ill patients. Therefore, this study aimed to identify clinical and metabolomic features associated with ICU mortality in patients receiving IMV or ECMO, as these remain incompletely characterized. **Methods**: The retrospective analysis included 30 ICU patients on IMV and 22 on ECMO. Metabolomic and proteomic profiling were performed using ultra-high-performance liquid chromatography coupled with high-resolution mass spectrometry (UHPLC-HRMS), and serum spectral analysis by Fourier-transform infrared spectroscopy (FTIRS). Significant variables were incorporated into multivariate logistic regression models, ranked by AIC, AUC, and statistical significance. Model performance was evaluated using stratified 5-fold cross-validation. Final models were adjusted for relevant demographic and clinical covariates. **Results**: The IMV cohort showed discriminatory FTIRS wavenumbers across all preprocessings, and 155 metabolites plus 14 proteins were significantly altered, with unadjusted models achieving mean AUCs above 0.9. The ECMO cohort showed discriminatory FTIRS wavenumbers in one preprocessing, and 15 metabolites plus 3 proteins were highlighted. FTIRS, metabolomic, and proteomic models reached mean AUCs of 0.967, 0.867, and 0.783, respectively, with lower stability during cross-validation. Adjustment for demographic and clinical covariates reduced model robustness. **Conclusions**: Stronger and more reproducible molecular signatures related to ICU mortality were observed in the IMV cohort, whereas the ECMO cohort showed reduced model stability, likely reflecting increased biological heterogeneity and small sample size. These findings support the utility of integrated omics for characterizing critical illness and outcome stratification, while reinforcing the need for validation in larger and independent cohorts.

## 1. Introduction

Invasive mechanical ventilation (IMV) and Extracorporeal membrane oxygenation (ECMO) became important rescue therapies for critically ill patients with refractory acute respiratory distress syndrome (ARDS), particularly during previous viral outbreaks such as H1N1 influenza and Middle East respiratory syndrome (MERS), and later during the COVID-19 pandemic [1,2,3,4,5]. Although ECMO demonstrated clinical benefit in selected patients with severe respiratory failure, mortality rates among critically ill patients requiring IMV and ECMO support remained substantially elevated [6]. In COVID-19-associated ARDS, reported mortality among ECMO-supported patients reached approximately 40% despite advances in intensive care management and updated clinical guidelines [7].

While several studies have characterized demographic and clinical predictors associated with poor outcomes in critically ill patients undergoing IMV or ECMO support, the underlying biological alterations associated with mortality are still not completely understood [8,9]. In particular, the systemic inflammatory response, immune dysregulation, oxidative stress, and metabolic dysfunction associated with severe respiratory failure and extracorporeal support may induce complex molecular alterations not fully captured by conventional clinical parameters alone [10].

In this context, omics-based approaches, including metabolomics, proteomics, and Fourier-transform infrared spectroscopy (FTIRS), may provide additional insight into the biological mechanisms and biomarkers associated with critical illness severity and mortality [11,12]. These techniques allow high-throughput characterization of metabolic, proteomic, and spectral features, potentially enabling identification of molecular signatures associated with clinical outcomes and ventilatory support-related biotrauma [13].

Although multiple machine learning approaches have been proposed for outcome prediction in critically ill patients [14], interpretable statistical models such as multivariate logistic regression may provide greater translational value in clinical settings by allowing quantitative assessment of the direction and magnitude of association between molecular variables and clinical outcomes [15]. Unlike some complex “black-box” models, these approaches facilitate biological interpretation of untargeted omics findings and may better support future personalized medicine strategies in intensive care [16,17,18,19].

Therefore, the aim of this study was to characterize mortality-associated molecular alterations in critically ill patients undergoing IMV and ECMO support through untargeted metabolomic, proteomic, and FTIRS analyses, while simultaneously evaluating the predictive performance and biological interpretability of multivariate logistic regression-based models.

## 2. Materials and Methods

### 2.1. Study Design

This retrospective exploratory study included critically ill ICU patients admitted to *Unidade Local de Saúde São José* (Lisbon, Portugal) [20]. These patients underwent invasive mechanical ventilation (IMV), with or without extracorporeal membrane oxygenation (ECMO) support. Patients were further stratified according to their ICU outcomes, namely into surviving and non-surviving groups.

A total of 52 patients were included, and four groups were created: patients requiring IMV that survived their ICU stay (IMV survivors; *n* = 15), patients requiring IMV that died during their ICU stay (IMV non-survivors; *n* = 15), patients requiring ECMO that survived their ICU stay (ECMO survivors; *n* = 13), and patients requiring ECMO that died during their ICU stay (ECMO non-survivors; *n* = 9).

Peripheral whole blood samples were collected from all patients according to the legal and ethical requirements (approval by the Hospital’s Ethics Commission: 1043/2021, 20 May 2020). Demographic and clinical data were retrieved from the hospital’s medical record system.

Metabolomic, proteomic, and FTIRS analyses were performed on the collected serum samples. The resulting data were used to evaluate significant differences between survivors and non-survivors within each ventilatory support group (IMV and ECMO), with the aim of developing outcome prediction models for each analytical platform.

### 2.2. Demographic, Clinical, and Laboratory Variables

A comprehensive set of demographic, clinical, and laboratory variables was extracted to characterize each patient group. Demographic variables included age, sex, and body-mass index (BMI). Clinical data comprised comorbidities such as arterial hypertension, diabetes mellitus, dyslipidemia, obesity, and chronic respiratory disease. Laboratory parameters measured at the time of blood sample collection included C-reactive protein (CRP), lactate, platelet count, lymphocyte count, neutrophil count, leukocyte count, hemoglobin, international normalized ratio (INR), bilirubin, and creatinine. Intervention-related variables included ICU length of stay, ICU day at sample collection, days on IMV, days on IMV at sample collection, days on ECMO, days on ECMO at sample collection, and ICU length of stay from sample collection to outcome (survival/death).

### 2.3. Serum Sample Preparation and UHPLC-HRMS Analysis

Whole blood stored in serum separator Vacutainer tubes was centrifuged at 756× *g* for 10 min, allowing serum extraction. The resultant serum was aliquoted and stored at −80 °C until subsequent analysis.

High-resolution mass spectrometry (HRMS) analysis was performed using an Elite ultra-high performance liquid chromatography (UHPLC) system, consisting of an Elite UHPLC HPG 1300 pump, equipped with two serially coupled independently controlled linear-drive pump heads, an Elite autosampler, and an Elite CSV column oven preheater (Bruker Daltonics GmbH & Co., Bremen, Germany). The UHPLC platform was coupled to an Impact II QqTOF mass spectrometer, equipped with an electrospray (ESI) source, enabling UHPLC-ESI-HRMS analysis.

#### 2.3.1. Metabolomics

Untargeted metabolomic analysis was performed according to the protocol previously described by Fonseca T. et al. [21]. Briefly, samples were analyzed by UHPLC-ESI-HRMS using both positive and negative electrospray ionization modes (ESI+ and ESI−). Two complementary chromatographic separations were employed: hydrophilic interaction liquid chromatography (HILIC) and reverse-phase chromatography (RP; C18).

Acquired MS data were calibrated using Data Analysis v5.1 (Bruker Daltonics GmbH & Co., Bremen, Germany), converted to mzXML format using ProteoWizard MSConvert [22], and processed using the XCMS server [23] for feature detection, retention-time correction, chromatographic alignment, feature grouping, quantification, and putative metabolite annotation. Processing parameters were optimized for UHPLC/MS data acquired on an Impact II Q-TOF platform. Samples were analyzed in triplicate, unless expressed otherwise, and resultant data were reduced to median values and further transformed to ln(x + 1) to reduce skewness and improve data distribution.

#### 2.3.2. Proteomics

The complete proteomics analysis workflow, including sample preparation, UHPLC-HRMS acquisition, and downstream data processing, has been previously described in detail by C. P. Von Rekowski et al. [12]. Briefly, serum proteins, analyzed in triplicate, were precipitated from 75 µL aliquots and processed using an in-house optimized filter-assisted sample preparation (FASP) protocol. This protocol included sequential protein denaturation, reduction, alkylation, and enzymatic digestion with trypsin prior to proteomic analysis [24,25].

Raw mass spectrometry data were processed using MaxQuant software v. 2.0.3.0 [26], employing the integrated Andromeda search engine [27], against the UniProt [28] database restricted to Homo sapiens protein sequences. Peptide and protein identifications were filtered at a 1% false discovery rate (FDR). Label-free quantification (LQF) intensities were calculated using the MaxLFQ algorithm implemented in MaxQuant [29].

The MaxQuant output data were subsequently processed in Perseus (version 1.6.15) using default parameters [30]. Contaminants, reverse hits, and proteins identified as “only identified by site” were excluded from further analysis. Protein intensity values were summarized by their median for each variable. Prior to statistical analysis, proteins presenting ≤30% zero values in at least one comparison group were retained for downstream analysis. The retained variables were transformed using ln(x + 1).

### 2.4. FTIRS

Serum samples were analyzed using FTIRS according to the protocol described by Araújo R. et al. [31]. Briefly, samples were analyzed in triplicate using 25 µL aliquots, diluted in distilled water at a 1:10 ratio, and pipetted onto a 96-well silicon plate. Following dehydration for 3.5 h in a desiccator, the plate was analyzed using an FTIR spectrometer (Vertex 70, Bruker, Mannheim, Germany) equipped with a High-Throughput Screening eXTension (HTS-XT) accessory (Bruker, Ettlingen, Germany).

Spectral data ranging from 400 to 4000 cm^−1^ were subsequently preprocessed with atmospheric compensation and baseline correction (Rubber-Band method), followed by additional preprocessing approaches including unit-vector normalization. For spectra processed solely with atmospheric compensation, a Savitzky–Golay derivative was additionally applied using a second-order polynomial over a 15-point window.

FTIRS spectral data acquired were summarized by their median value for each wavenumber. Prior to analyses, spectral matrices were scaled by multiplication factors of 10^4^ or 10^5^, as deemed appropriate to improve numerical interpretability and model stability.

### 2.5. Statistical Analysis

Baseline demographic, clinical, and laboratory characteristics were summarized using descriptive statistics. Continuous variables were reported as median with interquartile range (IQR), whereas categorical variables were expressed as counts and percentages. Group comparisons were performed using Mann–Whitney U tests for continuous variables and Chi-squared or Fisher’s exact test for categorical variables, depending on expected cell frequencies. Significant variables were further examined through univariate logistic regression to assess their predictive association with ICU mortality. Predictors achieving *p-*values < 0.250 were retained for subsequent adjustment in the multivariate analysis.

For FTIRS, metabolomic and proteomic datasets, differential feature screening between groups was conducted using Mann–Whitney U tests with further adjustment for multiple comparisons (e.g., Bonferroni, Holm, and False Discovery Rate). Significant features were then explored through univariate logistic regression, with variables achieving a *p-*value of less than 0.250 retained for multivariate testing, according to Hosmer–Lemeshow recommendations [32].

Multivariate logistic regression models were generated by exhaustive testing of all unique non-redundant predictor combinations. Candidate models were ranked according to Akaike Information Criterion (AIC), area under the curve (AUC), and overall statistical significance. Model performance was evaluated using both the full dataset (full model) and stratified 5-fold cross-validation, in which the dataset was divided into five subsets while preserving the distribution of survivors and non-survivors within each fold. For each iteration, the model was trained on four folds and validated on the remaining fold until all folds had been used once for validation.

Performance metrics, including AIC, AUC, AUC 95% confidence interval, and AUC *p-*value, were calculated independently for each cross-validation fold. The final reported values correspond to the mean of the fivefold-specific estimates. Additional model discrimination metrics, including classification accuracy, sensitivity, specificity, and their corresponding 95% confidence intervals, are provided in the Supplementary Material.

Statistical analyses were performed using IBM SPSS Statistics version 30 (IBM Corp., New York, NY, USA), RStudio version 2025.09.2 (Posit PBC, Boston, MA, USA), Python 3.12.4 through Jupyter Notebook version 7.2.2, and Unscrambler X version 10.4 (Camo Software AS, Oslo, Norway). R-based analyses, used mainly for univariate analysis, primarily employed the packages dplyr, ggplot2, ggrepel, tidyr, MASS, and ggforce. Python-based model generation and validation were conducted using the libraries pandas, numpy, statsmodels, and scikit-learn, including the StratifiedKFold function from the sklearn.model_selection module for stratified cross-validation procedures. Unscrambler X was used for spectral exploration and preprocessing. Statistical significance was defined as a *p*-value < 0.05.

**Figure 1:**
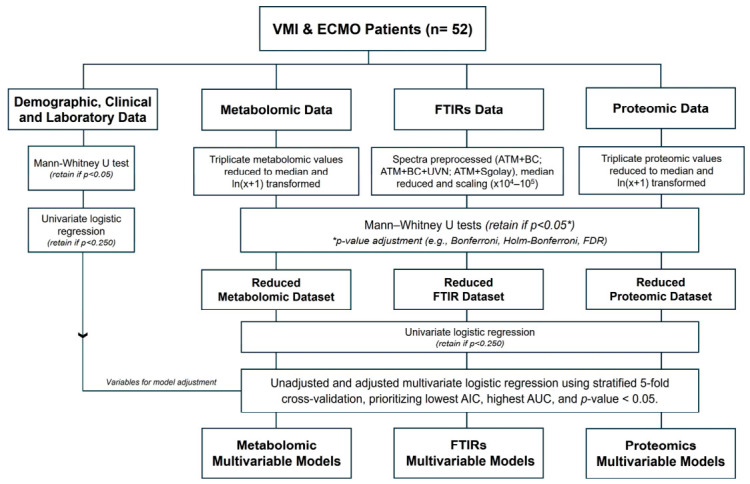
Schematic representation of the data analysis workflow. Raw data from metabolomic, FTIRS, and proteomic analyses were preprocessed, including triplicate aggregation (median values) and appropriate transformations (logarithmic, scaling, and spectral transformations). Univariate statistical analyses were performed to identify candidate features, which were subsequently included in the multivariate logistic regression models. Final model performance was evaluated using 5-fold stratified cross-validation, with selection based on the lowest AIC, highest AUC, and a *p*-value < 0.05.

A schematic overview of the analytical workflow and sequential decision-making process adopted in this study is presented in Figure 1.

## 3. Results

The results are presented according to the two ventilatory support groups, namely IMV and ECMO. Within each cohort, survivors and non-survivors were compared considering demographic, clinical, laboratory, FTIRS, metabolomic, and proteomic data, followed by predictive model development.

### 3.1. Demographic, Clinical, and Laboratory Data

Baseline characteristics of patients undergoing IMV are summarized in Table 1. Compared with non-survivors, IMV survivors were significantly younger (*p* = 0.037). Arterial hypertension was more frequently observed among non-survivors (*p* = 0.025). Regarding laboratory parameters, non-survivors presented higher lactate levels (*p* = 0.041) and higher INR values (*p* = 0.041). No intervention-related variables significantly differed between groups. APACHE II scores and SOFA scores at ICU admission did not differ significantly between survivors and non-survivors (Table S1). However, SOFA scores at the time of sample collection were significantly higher in non-survivors (*p* = 0.0047), indicating greater organ dysfunction at the time of metabolomic profiling. Univariate logistic regression analysis (Table 1) showed that increasing age was associated with a higher likelihood of ICU mortality (crude OR = 1.26, *p* = 0.052). Arterial hypertension (crude OR = 6.00, *p* = 0.032), lactate levels (crude OR = 3.438, *p* = 0.071), and INR (crude OR = 1.147 × 10^3^, *p* = 0.038) were also associated with increased mortality risk.

Regarding the ECMO cohort, baseline characteristics are summarized in Table 2. Non-survivors were significantly older than ECMO survivors (*p* = 0.007). No other clinical or laboratory variables differed significantly between groups, except for the duration of IMV at the time of sample collection, which was higher in non-survivors (*p* = 0.030). No significant differences were observed between survivors and non-survivors regarding APACHE II scores or SOFA scores, either at ICU admission or at the time of sample collection (Table S2). Univariate logistic regression (Table 2) indicated that increasing age was associated with higher odds of ICU mortality (crude OR = 1.331, *p* = 0.048), as well as longer duration of IMV at the time of sampling (crude OR = 1.538, *p* = 0.048), suggesting that prolonged exposure to invasive ventilatory support may be associated with worse outcomes in ECMO patients.

Overall, age emerged as a consistent predictor of ICU mortality across both ventilatory support strategies, whereas additional clinical predictors differed between cohorts. These variables were subsequently considered as candidate adjustment covariates for the multivariable predictive models.

### 3.2. FTIRS Spectral Analysis

#### 3.2.1. IMV Cohort

**Figure 2:**
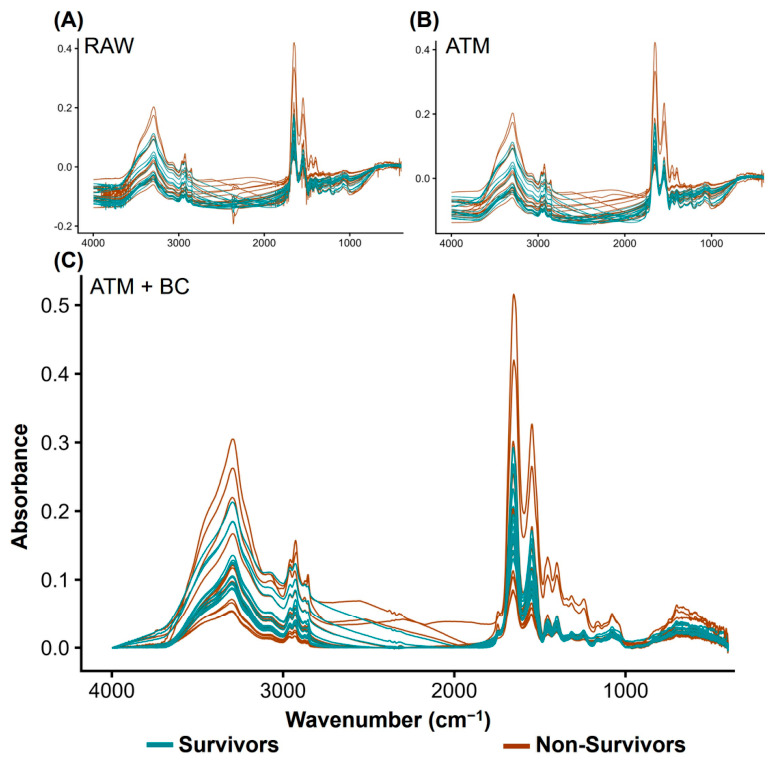
IMV patients’ raw FITRS spectra and spectra after ATM and ATM + BC corrections. The patients’ serum spectra are displayed according to different pre-processing approaches: (**A**) raw spectra; (**B**) Atmospheric (ATM) compensation for CO_2_ and H_2_O interference; and (**C**) Atmospheric and Baseline (ATM + BC) correction, the latter performed using the Rubberband method. The y-axis represents the absorbance values of each wavenumber of each patient sample, while the x-axis represents the wavenumber in descending order. Individual patient spectra are colored according to survival status (dark blue: survivors; dark orange: non-survivors).

Raw FTIRS spectra were pre-processed using atmospheric correction (ATM) and baseline correction (BC) to minimize environmental interference and baseline drifts (Figure 2).

**Figure 3:**
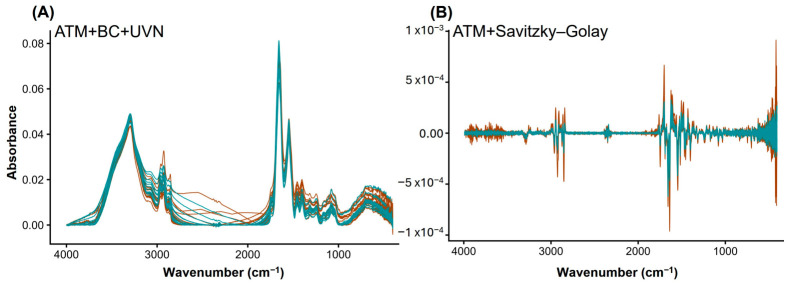
IMV patients’ raw FITRS spectra with ATC + BC + UVN and ATM + Savitzky–Golay corrections. The patients’ spectra are displayed according to different pre-processing approaches: (**A**) Atmospheric (ATM) compensation for CO_2_ and H_2_O interference and unit-vector normalization (UVN); (**B**) ATM and Savitzky–Golay preprocessing with a 2nd derivative order with 15 points. Individual patient spectra are colored according to survival status (dark blue: survivors; dark orange: non-survivors).

Other preprocessing techniques, such as unit-vector normalization (UVN) and the Savitzky–Golay filter, were tested (Figure 3). The impact of these spectral preprocessing methods (ATM + BC; ATM + BC + UVN and ATM + Savitzky–Golay) was further evaluated by comparing survivor and non-survivor group absorbances across all wavenumbers. Ultimately, the analysis focused on the 3400–2800 cm^−1^ and 1800–600 cm^−1^ regions, which contain the most informative biochemical signatures in biological samples and less noise.

**Figure 4:**
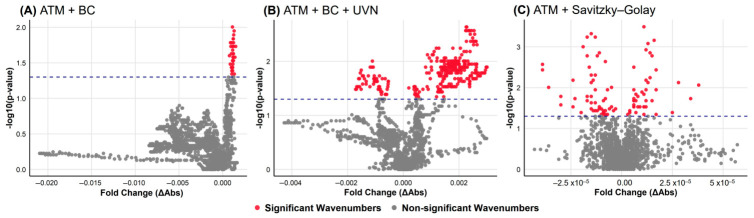
Volcano plots depicting significantly different wavenumbers between IMV survivors and non-survivors, using different preprocessing methods: (**A**) Atmospheric and Baseline (ATM + BC) correction; (**B**) Atmospheric (ATM) compensation and unit-vector normalization (UVN); and (**C**) ATM correction followed by Savitzky–Golay smoothing. Each dot represents an individual wavenumber from patients’ FTIRS spectra. The x-axis shows the difference in absorbances between groups (delta absorbance, ΔAbs = median absorbance of non-survivors–median absorbance of survivors), while the y-axis represents the raw *p*-values of the comparison between the absorbances of these groups, transformed as −log_10_(*p*-value) (dashed blue line indicates the significance threshold).

Pairwise comparisons between survivors and non-survivors using the ATM + BC preprocessing led to the identification of 34 significantly different wavenumbers between groups. However, after multiple testing correction, none remained significant. This outcome was expected, as the number of statistical tests performed (1867 wavenumbers) greatly exceeded the number of patients in each group, contributing to relatively low raw *p*-values and resulting in a strong penalization of the adjusted *p*-values. For the purposes of the study, raw *p*-values were considered and are displayed in the volcano plot shown in Figure 4A.

The significant wavenumbers were distributed across several spectral regions, including around the 1360 cm^−1^ region (6 variables), 1350 cm^−1^ region (6 variables), 1010 cm^−1^ region (4 variables), 1000 cm^−1^ region (7 variables), 990 cm^−1^ region (8 variables), as well as the 980 cm^−1^ region (3 variables). The ATM + BC + UVN preprocessing (Figure 4B) retrieved a higher number, specifically 328 wavenumbers. These variables were clustered within several spectral regions, including around 3296–3241 cm^−1^, 1624–1575 cm^−1^, 1507–1313 cm^−1^, and 1011–989 cm^−1^, with an additional isolated wavenumber at 979 cm^−1^. Using the Savitzky–Golay preprocessing, 93 wavenumbers stayed significantly different between survivors and non-survivors (Figure 4C). These variables were also distributed across several spectral regions, including the high-wavenumber region around 3300–3200 cm^−1^, and the 2970–2820 cm^−1^, 1694–1409 cm^−1^, 1324–1000 cm^−1^, and 995–618 cm^−1^ regions.

**Figure 5:**
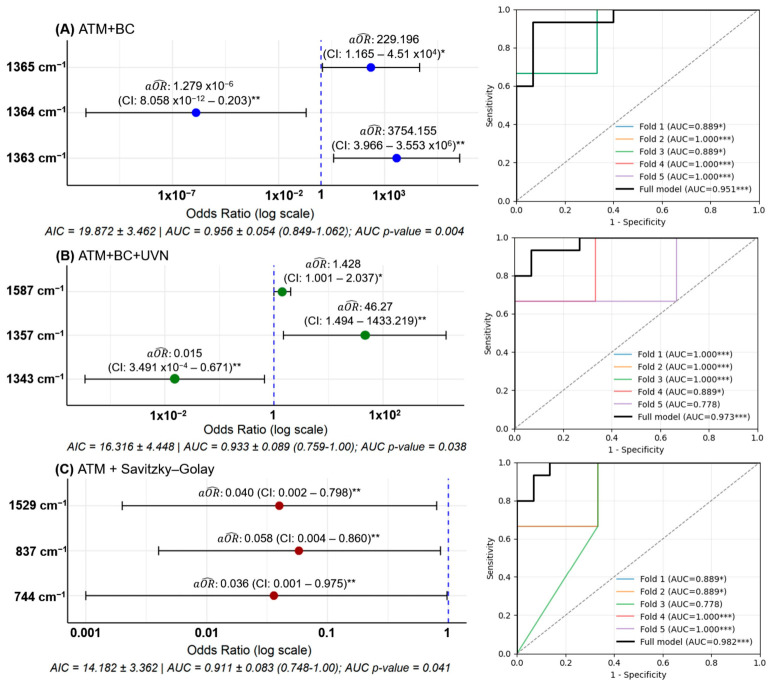
Multivariate logistic regression models for mortality prediction in IMV patients derived from different FTIRS spectral preprocessing strategies: (**A**) ATM + BC, (**B**) ATM + BC + UVN, and (**C**) ATM + Savitzky–Golay. Forest plot showing adjusted odds ratio ($a\hat{OR}$) and corresponding 95% confidence intervals estimated from the full dataset. The x-axis is displayed on a log_10_ scale for improved visualization. Performance metrics (AIC and AUC) are presented as mean ± standard deviation and were estimated using 5-fold stratified cross-validation (AIC, AUC, AUC 95% confidence interval, AUC *p*-value). For each model, receiver operating characteristic (ROC) curves are presented for all folds obtained through 5-fold stratified cross-validation (blue, yellow, green, red and purple lines), together with the overall model performance (“Full model”, black line), depicted as a bold black line. Statistical significance is indicated as * *p* < 0.05, ** *p* < 0.01, and *** *p* < 0.001.

To further explore the potential predictive value of the identified spectral variables, univariate logistic regression analyses were performed. Each wavenumber was individually tested for its association with patient outcomes. Regarding the ATM + BC, ATM + BC + UVN, and ATM + Savitzky–Golay preprocessing, the univariate analysis identified 31, 324, and 92 candidate wavenumbers, respectively, for subsequent inclusion in multivariate modeling. The multivariate models generated from each preprocessing approach were evaluated using stratified 5-fold cross-validation (Figure 5; complete model metrics provided in Table S1).

The multivariate model derived from ATM + BC preprocessing (Figure 5A) showed robust predictive performance (mean AUC = 0.956 ± 0.054). The model included three adjacent wavenumbers, specifically 1365, 1364, and 1363 cm^−1^, highlighting the relevance of this spectral band.

The multivariate model derived from ATM + BC + UVN preprocessing (Figure 5B) also showed robust predictive performance (mean AUC = 0.933 ± 0.089), with a lower AIC compared to the ATM + BC model, indicating improved model fit. The model included wavenumbers at 1587, 1357, and 1343 cm^−1^, spanning distinct spectral regions. These variables likely reflect complementary biochemical information, particularly within protein-related (amide II) and lipid-associated bands, contributing jointly to the prediction of patient outcome.

The multivariate model derived from Savitzky–Golay preprocessing (Figure 5C) showed good predictive performance (AUC = 0.911 ± 0.083; *p* = 0.041) and the lowest AIC among all three models, indicating the best balance between model fit and complexity. The model included wavenumbers 1529, 837, and 744 cm^−1^, spanning both protein-associated (amide II) and fingerprint spectral regions. These findings suggest that this preprocessing approach captures a more distributed and biologically relevant spectral signature associated with patient outcome.

#### 3.2.2. ECMO Cohort

**Figure 6:**
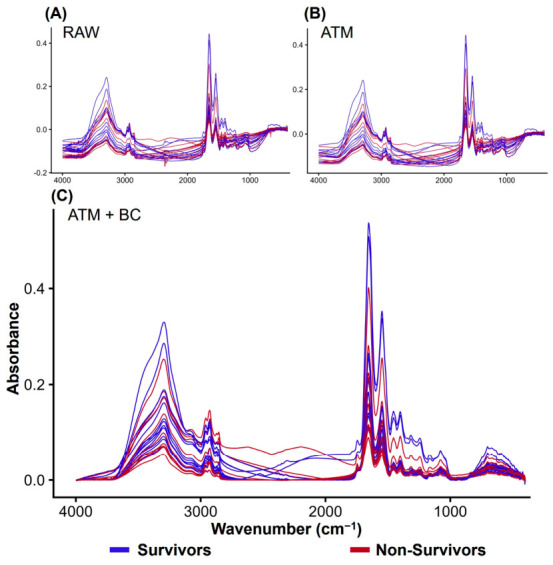
ECMO patients’ raw FTIRS spectra and spectra after ATM and ATM + BC corrections. The patients’ spectra are displayed according to different pre-processing approaches: The patients’ serum spectra are displayed according to different pre-processing approaches: (**A**) raw spectra; (**B**) Atmospheric (ATM) compensation for CO_2_ and H_2_O interference; and (**C**) Atmospheric and Baseline (ATM + BC) correction, the latter performed using the Rubberband method. The y-axis represents the absorbance values of each wavenumber of each patient sample, while the x-axis represents the wavenumber in descending order. Individual patient spectra are colored according to survival status (blue: survivors; red: non-survivors).

Using the same preprocessing strategy applied to the IMV cohort, raw FTIRS spectra from ECMO patients were visually inspected and subsequently corrected using the ATM and BC procedures (Figure 6).

**Figure 7:**
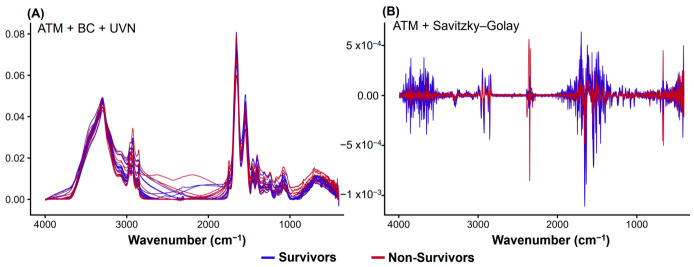
ECMO patients’ raw FITRS spectra with ATC + BC + UVN and ATM + Savitzky–Golay corrections. The patients’ spectra are displayed according to different pre-processing approaches: (**A**) Atmospheric (ATM) compensation for CO_2_ and H_2_O interference followed by unit-vector normalization (UVN); and (**B**) ATM correction followed by Savitzky–Golay preprocessing using a second-order derivative with a 15-point smoothing window. Individual patient spectra are colored according to survival status (blue: survivors; red: non-survivors).

The same additional preprocessing approaches described for the IMV cohort (ATM + BC + UVN and ATM + Savitzky–Golay) were applied to the ECMO spectra (Figure 7), and the resulting datasets were subsequently compared between survivors and non-survivors within the predefined informative spectral regions (i.e., the 3400–2800 cm^−1^ region and 1800–600 cm^−1^ region).

**Figure 8:**
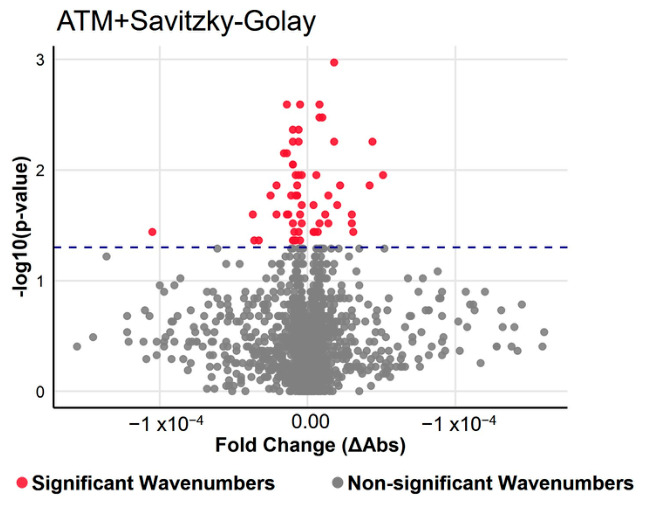
Volcano plot depicting significantly different wavenumbers between ECMO survivors and non-survivors, using ATM correction followed by Savitzky–Golay smoothing. Each point represents an individual wavenumber from the FTIRS spectra. The x-axis shows the difference in median absorbance between groups (ΔAbs = median absorbance of non-survivors—median absorbance of survivors), while the y-axis represents -log_10_ transformed unadjusted *p*-values from group comparison. The dashed blue line indicates the nominal significance threshold.

Regarding the pairwise comparison between ECMO survivors and non-survivors, no significantly different wavenumbers were identified for the ATM + BC and ATM + BC + UVN preprocessed datasets. In contrast, the Savitzky–Golay-processed spectra revealed 60 wavenumbers with nominal statistical significance. After correction for multiple comparisons, none of these variables remained below the significance threshold; therefore, due to the exploratory nature of these analyses, raw *p*-values were considered for logistic regression analysis (Figure 8).

**Figure 9:**
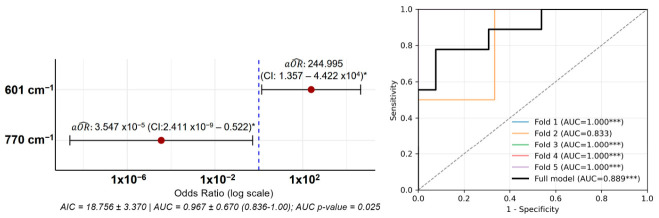
Multivariate logistic regression models for mortality prediction in ECMO patients derived from FTIRS spectral ATM + Savitzky–Golay preprocessing. Forest plot showing adjusted odds ratio ($a\hat{OR}$) and corresponding 95% confidence intervals estimated from the full dataset. The x-axis is displayed on a log_10_ scale for improved visualization. Performance metrics (AIC and AUC) are presented as mean ± standard deviations and were estimated using 5-fold stratified cross-validation (AIC, AUC, AUC 95% confidence interval, AUC *p*-value). Receiver operating characteristic (ROC) curves are presented for all folds obtained through 5-fold stratified cross-validation (blue, yellow, green, red and purple lines), together with the overall model performance (“Full model”, black line), depicted as a bold black line. Statistical significance is indicated as * *p* < 0.05, and *** *p* < 0.001.

After scaling of the Savitzky–Golay FTIRS dataset (×10^5^) to ensure numerical stability, univariate logistic regression retained 59 wavenumbers for multivariate modeling (Figure 9; complete model metrics provided in Table S2). Among all models tested with combinations of up to five wavenumbers, the best-performing model for the Savitzky–Golay achieved mean AUCs of 0.967 ± 0.067. This model included the wavenumbers 601 cm^−1^ and 770 cm^−1^, with 601 cm^−1^ showing an adjusted odds ratio > 1 and 770 cm^−1^ showing an adjusted odds ratio < 1.

### 3.3. Metabolomics

#### 3.3.1. IMV Cohort

The metabolites identified through metabolomics analysis were first subjected to pairwise comparisons between survivors and non-survivors. A total of 155 features showed significant differences between groups and remained significant after FDR correction. These were therefore selected for univariate logistic regression analysis. All 155 features remained significant in the univariate logistic regression and were subsequently included in the multivariate modeling.

**Figure 10:**
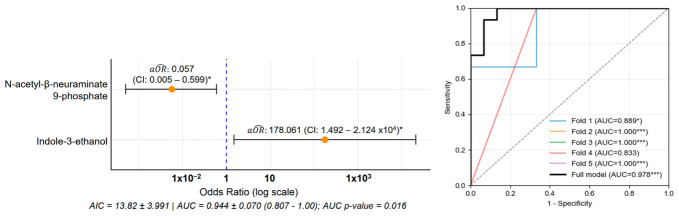
Best-performing multivariate regression model derived from metabolomic data for mortality prediction in IMV patients. Forest plot showing adjusted odds ratio ($a\hat{OR}$) and corresponding 95% confidence intervals estimated from the full dataset. The x-axis is displayed on a log_10_ scale for improved visualization. AIC and AUC are presented as mean ± standard deviation, estimated using 5-fold stratified cross-validation. Receiver operating characteristic (ROC) curves are presented for all folds obtained through 5-fold stratified cross-validation (blue, yellow, green, red and purple lines), together with the overall model performance (“Full model”), depicted as a bold black line. Statistical significance is indicated as * *p* < 0.05, and *** *p* < 0.001.

Due to the high dimensionality of the dataset, multivariate models were limited to combinations of up to four variables to ensure computational tractability. The best-performing multivariate model for predicting IMV mortality based on metabolomic features included N-acetyl-β-neuraminate 9-phosphate ($a\hat{OR}$ = 0.057; 95% CI: 0.005–0.599; *p* = 0.017) and indole-3-ethanol ($a\hat{OR}$ = 178.061; 95% CI: 1.492–2.124 × 10^4^; *p* = 0.034), showing strong predictive performance (mean AUC = 0.944 ± 0.070) (Figure 10; complete model metrics provided in Table S3).

#### 3.3.2. ECMO Cohort

A total of 15 metabolites differed significantly between ECMO survivors and non-survivors. In contrast to the IMV cohort, none of these metabolites remained significant after correction for multiple comparisons. For exploratory purposes, the 15 metabolites were subsequently evaluated using univariate logistic regression, revealing crude associations with ICU mortality for 12 features. Multivariate logistic regression analysis yielded only a limited number of statistically significant models, generally composed of one or two metabolites. However, none of these models showed significant discriminative performance, as reflected by non-significant AUC values.

**Figure 11:**
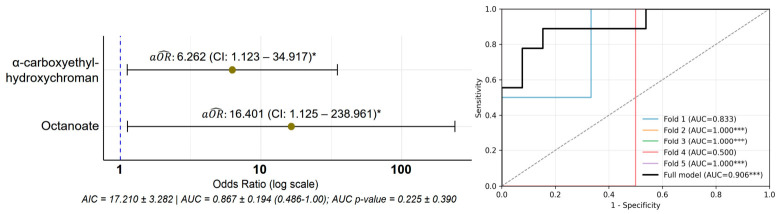
Best-performing multivariate regression model derived from metabolomic data for mortality prediction in ECMO patients. Forest plot showing adjusted odds ratio ($a\hat{OR}$) and corresponding 95% confidence intervals estimated from the full dataset. The x-axis is displayed on a log_10_ scale for improved visualization. AIC and AUC are presented as mean ± standard deviation, estimated using 5-fold stratified cross-validation. Receiver operating characteristic (ROC) curves are presented for all folds obtained through 5-fold stratified cross-validation (blue, yellow, green, red and purple lines), together with the overall model performance (“Full model”), depicted as a bold black line. Statistical significance is indicated as * *p* < 0.05, and *** *p* < 0.001.

Among the evaluated models, the combination of α-carboxy-ethylhydroxychroman and octanoate showed the highest predictive performance. Both metabolites were independently associated with ICU mortality, with adjusted odds ratios of 6.262 (95% CI: 1.123–34.917; *p* = 0.036) and 16.401 (95% CI: 1.125–238.961; *p* = 0.041), respectively (Figure 11; complete model metrics provided in Table S4). This model achieved a mean AUC of 0.867 ± 0.194; however, the average AUC *p*-value was non-significant (*p* = 0.225), indicating that, despite its apparently promising performance, the model lacked robust discriminative capacity in this limited-size cohort.

### 3.4. Proteomics

#### 3.4.1. IMV Cohort

A total of 14 proteomic features were significantly different between survivor and non-survivor patients undergoing IMV. However, no features remained statistically significant after multiple testing correction. All 14 variables showed a crude association with ICU mortality in the univariate logistic regression and were therefore selected for inclusion in the multivariate analysis. All proteins exhibited odds ratios below 1, suggesting a consistent inverse association with the outcome.

**Figure 12:**
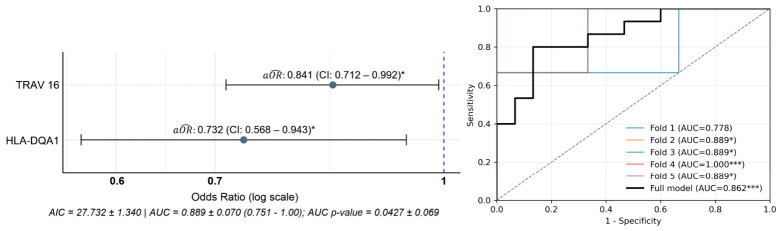
Best-performing multivariate regression model derived from proteomics data for mortality prediction in IMV patients. Forest plot showing adjusted odds ratio ($a\hat{OR}$) and corresponding 95% confidence intervals estimated from the full dataset. The x-axis is displayed on a log_10_ scale for improved visualization. AIC and AUC are presented as mean ± standard deviation, estimated using 5-fold stratified cross-validation. Receiver operating characteristic (ROC) curves are presented for all folds obtained through 5-fold stratified cross-validation (blue, yellow, green, red and purple lines), together with the overall model performance (“Full model”), depicted as a bold black line. Statistical significance is indicated as * *p* < 0.05, and *** *p* < 0.001.

The best-performing multivariate logistic regression model included the protein T cell receptor alpha variable 16 (TRAV 16) and MHC class II antigen DQA1 (HLA-DQA1), both showing a statistically significant inverse association with the outcome ($a\hat{OR}$ = 0.841; 95% CI: 0.712–0.992; *p* = 0.045, and $a\hat{OR}$ = 0.732; 95% CI: 0.568–0.943; *p* = 0.016, respectively). Average model performance achieved a mean AUC of 0.889 ± 0.070; however, significance was not consistent across all folds (Figure 12; complete model metrics provided in Table S5).

#### 3.4.2. ECMO Cohort

**Figure 13:**
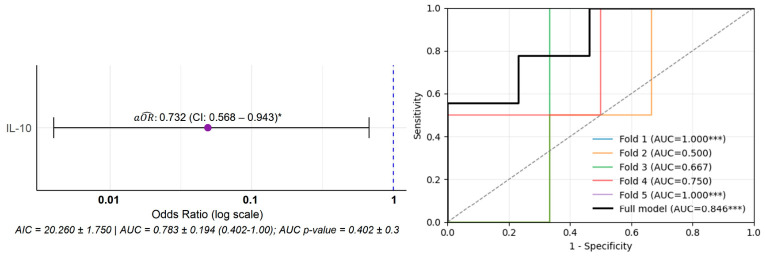
Best-performing multivariate regression model derived from proteomics data for mortality prediction in ECMO patients. Forest plot showing adjusted odds ratio ($a\hat{OR}$) and corresponding 95% confidence intervals estimated from the full dataset. The x-axis is displayed on a log_10_ scale for improved visualization. AIC and AUC are presented as mean ± standard deviation, estimated using 5-fold stratified cross-validation. Receiver operating characteristic (ROC) curves are presented for all folds obtained through 5-fold stratified cross-validation, together with the overall model performance (“Full model”), depicted as a bold black line. Statistical significance is indicated as * *p* < 0.05, and *** *p* < 0.001.

For the ECMO cohort, 3 proteins were significantly different between survivors and non-survivors. All of them remained significantly associated with ICU mortality in the univariate logistic regression analysis. However, in the multivariate modeling step, only a single model was deemed significant, including interleukin-10 (IL-10; A0A286YEX3) and an adjusted odds ratio of 0.049 (95% CI: 0.004–0.673; *p* = 0.024). However, the model showed limited discriminative performance, with a non-significant average AUC of 0.783 ± 0.194 (Figure 13; complete model metrics provided in Table S6).

### 3.5. Multivariate Models Adjusted for Demographic and Clinical Variables

The best-performing omics-derived models were subsequently adjusted for significant demographic, clinical, and laboratory covariates identified in Section 3.1.

Adjusted FTIRS-derived models exhibited reduced numerical stability during cross-validation (Table 3; complete model metrics provided in Table S7). In the IMV cohort, the ATM + BC model failed in four of five folds because of singular matrix errors, consistent with quasi-separation and difficulties in estimating reliable coefficients. Similar issues were observed when the model was fitted to the full dataset. For the ATM + BC + UVN model, valid estimates were obtained in only two of five folds, although not significant. Likewise, fitting the adjusted model to the full dataset resulted in non-convergence and unstable coefficient estimates. The ATM + Savitzky–Golay model yielded a valid estimate in only one validation fold, although a stable model could be fitted using the full dataset; none of the coefficients were statistically significant (Table 3). Collectively, these findings highlight the importance of cross-validation in assessing model robustness and generalizability.

Within the ECMO cohort, the ATM + Savitzky–Golay was the only FTIRS preprocessing that generated a valid multivariate model. After adjustment for age and days under IMV at the time of serum sampling, two of five validation folds failed during cross-validation, and the model fitted to the full dataset yielded non-significant coefficients (Table 3).

Regarding the metabolomic and proteomic analyses, adjustment for significant clinical and demographic covariates resulted in the models presented in Table 4 (complete model metrics provided in Table S8). For the adjusted metabolomic IMV model, substantial numerical instability was observed after inclusion of the significant clinical and demographic covariates. During 5-fold cross-validation, three of the five folds failed because of singular matrix errors, while the remaining folds converged only partially. Among the valid folds, coefficient estimates were highly unstable, with extreme or non-interpretable odds ratios, undefined confidence intervals, and inconsistent *p*-values. Although the model fitted to the complete dataset formally converged and yielded apparently perfect classification metrics, the resulting coefficients were either infinite, null, or otherwise non-informative, indicating quasi-complete separation and severe overfitting rather than a robust predictive solution.

For the adjusted metabolomic model in the ECMO cohort, logistic regression displayed marked numerical instability during internal validation. Two of the five cross-validation folds failed due to singular matrix errors, while the remaining folds produced extreme coefficient estimates, boundary confidence intervals, and uniformly non-significant coefficient *p*-values. Although some folds achieved apparently high discrimination (AUCs ranging from 0.833 to 1.00), these results should be interpreted cautiously, as they likely reflect overfitting driven by the small sample size and sparse outcome structure rather than true predictive robustness. Importantly, when fitted to the complete dataset, the model failed to converge, and no stable full-model estimates could be obtained. Overall, these findings indicate that the adjusted metabolomic model lacked sufficient numerical stability and reproducibility for reliable inference in the ECMO cohort.

For the adjusted proteomic model in the IMV cohort, inclusion of the significant demographic, clinical, and laboratory covariates resulted in numerically stable logistic regression models across all five cross-validation folds, with no convergence failures or singular matrix errors. Discriminative performance was moderate to high, with fold-specific AUC values ranging from 0.778 to 1.000. When fitted to the complete dataset, the model achieved an apparent AUC of 0.916 and an overall classification accuracy of 76.7% (Supplementary Table S8). However, none of the adjusted covariates or proteomic variables remained independently significant in the full model, and several estimates were accompanied by wide confidence intervals, indicating limited precision likely related to the small sample size and model complexity. Overall, this model showed greater numerical robustness during internal validation than the adjusted FTIRS and metabolomic models, although coefficient stability remained limited.

For the adjusted proteomic model in the ECMO cohort, logistic regression showed partial numerical stability during internal validation, with one of the five cross-validation folds failing due to singular matrix error. The remaining folds converged successfully, yielding variable predictive performance, with AUC values ranging from 0.50 to 1.00. However, coefficient estimates were highly imprecise, with extremely wide confidence intervals and no statistically significant predictors across folds. When fitted to the complete dataset, the model achieved an apparent AUC of 0.974 and an overall classification accuracy of 90.9% (Supplementary Table S8); nevertheless, none of the adjusted coefficients reached statistical significance, indicating limited inferential reliability. These findings suggest that, despite apparently strong discrimination, the adjusted proteomic model was likely affected by overfitting and small sample instability and therefore should be interpreted with caution.

As age was systematically associated with the outcome, correlation analysis with the metabolites present in the models was performed (Figure S1). Out of the 4 metabolites described, only indole-3-ethanol showed a moderate association with age (Spearman ρ = 0.495; *p* = 0.006).

A final comparison between significant demographics/clinical variables and significant metabolomic variables was performed to observe if there was an incremental prognostic value of omics features over standard clinical predictors such as age, lactate, and INR in the IMV cohort, and age and days with IMV at sample collection in the ECMO cohort. Compared with demographic/clinical models (Supplementary Table S10), metabolomic models consistently showed superior predictive performance in both cohorts. In the IMV cohort, the metabolomic model markedly improved discrimination (AUC 0.944 vs. 0.778), reduced model complexity as reflected by a substantially lower AIC (13.8 vs. 31.6), and increased accuracy (90.0% vs. 80.0%). Similar improvements were observed in the ECMO cohort, where metabolomic features yielded higher AUC (0.867 vs. 0.833), lower AIC (17.2 vs. 21.8), and better accuracy, sensitivity, and specificity. These findings indicate that metabolomic biomarkers may provide prognostic information beyond conventional demographic and clinical variables.

## 4. Discussion

This study addresses the potential of omics-based tools, including mass spectrometry platforms applied to metabolomics and proteomics, as well as FTIRS for serum spectral profiling, to explore the biological impact of ventilatory support therapies in two ICU cohorts.

Within each cohort, survivors and non-survivors were generally comparable regarding baseline demographic and clinical characteristics, with only a limited number of variables differing between outcome groups. In both the IMV and ECMO cohorts, deceased patients were older than survivors. In the IMV cohort, non-survivors also showed a higher prevalence of arterial hypertension, together with higher lactate and INR values on the day of sample collection. In the ECMO cohorts, non-survivors had undergone longer duration of IMV at the time of sampling, which may represent a potential confounding factor related to disease severity or prolonged critical illness. These clinical differences observed between survivors and non-survivors were consistent with previous critical care literature. Advanced age had been associated with increased mortality in severe respiratory failure, including in patients requiring IMV [5] and ECMO [7]. Elevated lactate levels may reflect circulatory dysfunction [33] and global tissue hypoperfusion [34], whereas prolonged INR may indicate coagulation disturbances and greater systemic severity [35]. Although associated with mortality at baseline, these covariates did not consistently improve the predictive performance of the omics-derived models and were frequently associated with reduced robustness following internal validation, as reflected by poorer model stability and generalizability.

Regarding illness severity, IMV non-survivors presented significantly higher SOFA scores at the time of sample collection, indicating greater organ dysfunction than survivors at the time of metabolomic profiling, despite the median interval between sample collection and death being approximately 9 days.

Overall, the IMV cohort showed a stronger and more consistent omics signal compared with the ECMO cohort across all analytical platforms. In the FTIRS analysis, the IMV cohort yielded significant wavenumbers across all preprocessing strategies, whereas in the ECMO cohort significant discrimination was only observed under specific preprocessing conditions (ATM correction and Savitzky–Golay), with a lower number of discriminant wavenumbers overall. A similar pattern was observed in metabolomics, where 155 metabolites remained significant after FDR correction in the IMV cohort, compared with only 15 differentially abundant metabolites in the ECMO cohort, which did not retain significance after multiple testing correction and were only considered under exploratory thresholds. Proteomic analysis followed the same trend, with 14 significantly associated proteins identified in the IMV cohort versus only three in the ECMO cohort. FTIRS-based models retained discriminatory capacity across both cohorts, although with a reduced number of discriminative features in ECMO patients, whereas metabolomic and proteomic signatures lost statistical discriminative ability in the ECMO cohort. Collectively, these findings suggest a reduced detectable biological signal in the ECMO cohort, likely underpowered by the smaller sample size and higher diversity of metabolic profiles.

A consistent finding across platforms was that inclusion of significant demographic, clinical, and laboratory covariates did not improve model performance and frequently reduced numerical stability during internal validation. This pattern is compatible with quasi-separation and over-parameterization in relation to sample size [36]. Taken together, these findings indicate that adding conventional covariates to omics-derived signatures did not systematically strengthen prediction and, in several instances, impaired generalizability. This likely reflects the combination of small-sample size/low-event-counts, and correlation between clinical variables and omics features, which can destabilize maximum-likelihood logistic regression models. Consequently, the stratified cross-validation results are able to highlight that apparent full-sample performance should be interpreted cautiously, as it may substantially overestimate true external predictive utility [37].

From a translational perspective, the identified FTIRS spectral signatures and omics-based molecular profiles represent candidate patterns that, after analytical and clinical validation, could potentially support rapid biological assessment and risk stratification of critically ill patients. FTIRS offers advantages in terms of rapid acquisition, minimal sample preparation, and global biochemical fingerprinting, whereas metabolomics and proteomics provide deeper molecular characterization of disease-related alterations. The integration of these complementary approaches with clinical variables and predictive models may contribute to future precision medicine strategies in critical care, although validation in larger independent cohorts remains essential before clinical implementation.

### 4.1. FTIRS

Regarding the unadjusted multivariate modeling of FTIRS data, the analysis identified candidate wavenumbers with potential to discriminate survivors from non-survivors in both the IMV and ECMO cohorts. Overall, these models had a favorable discriminatory performance, with robust AUC values across most preprocessing strategies. The main exception was the ECMO cohort, in which the limited sample size likely constrained statistical power and reduced the ability to consistently detect significantly discriminant spectral variables across all preprocessing approaches.

In the IMV cohort, the ATM + BC preprocessing approach identified the band ~1364 cm^−1^ in the best-performing unadjusted multivariate model, associated with contributions from δs(CH3), ν(C=O), C–CH3 and γs(CH3) likely from lipids and proteins, which may reflect alterations in serum biochemical composition involving lipid- and protein-associated structures. This spectral region may be linked to the metabolic disturbances occurring during critical illness [38,39,40].

When preprocessing data with UVN, the best unadjusted model comprised the 1357 and 1343 cm^−1^, and the 1587 cm^−1^ band. The 1587 cm^−1^ band lies closer to the spectral region between ~1580 and 1590 cm^−1^, likely reflecting a complex overlap of aromatic ring C-C stretching vibrations [40] and protein-related Amide II contributions [41].

Although the Savitzky–Golay model did not substantially outperform alternative preprocessing strategies in terms of overall discrimination, it had notable consistency in effect direction across analytical levels. Specifically, for the wavenumbers 1529, 837 and 744 cm^−1^, univariate comparisons showed consistent group-wise differences that were preserved in the multivariable logistic regression, with odds ratios maintaining the same directional association with mortality. This contrasts with the behavior observed in other preprocessing pipelines, where multivariable coefficient estimates were more sensitive to collinearity and occasionally diverged from univariate trends. As Savitzky–Golay preprocessing involves derivative transformation, the resulting spectra reflect changes in signal slope rather than absolute absorbance, with regions of decreasing absorbance represented as negative values (e.g., reflecting more the biochemical composition rather than absolute absorbance differences between metabolites). While this enhances detection of overlapping features, it complicates direct comparison with raw spectral intensities. Nevertheless, these findings suggest that Savitzky–Golay preprocessing may provide improved preservation of underlying spectral signal structure, leading to greater coherence between univariate and multivariate inference rather than enhanced predictive performance per se.

Regarding the ECMO cohort, due to its limited sample size, it was more challenging to find significantly distinct variables between the two outcome groups. Nonetheless, with the Savitzky–Golay preprocessing, it was possible to develop a multivariate model comprising wavenumbers within the fingerprint region, namely 770 and 601 cm^−1^. As observed in the IMV cohort, this preprocessing approach maintained consistency in the direction of association of these wavenumbers throughout the statistical analysis, with 601 cm^−1^ showing a positive association with mortality and 770 cm^−1^ an inverse association.

Collectively, these models may reflect coordinated alterations in serum biochemical composition associated with clinical outcomes under IMV and ECMO support. These candidate spectral features warrant further investigation in larger cohorts to determine their reproducibility and potential contribution to outcome prediction.

### 4.2. Metabolomics

Metabolomic analysis in the IMV cohort identified a multivariable model comprising *N*-acetyl-β-neuraminate 9-phosphate (Neu5Ac-9P) and indole-3-ethanol, which indicated consistent directionality and statistical significance across cross-validation folds and in the full dataset. Specifically, Neu5Ac-9P was inversely associated with mortality, whereas indole-3-ethanol showed a positive association. Neu5Ac-9P is involved in sialic acid metabolism [42], a pathway closely related to glycoprotein synthesis, immune modulation [43], and endothelial function [44]. Its inverse association with mortality may reflect preserved metabolic or structural integrity in survivors. One plausible hypothesis is that lower levels of this intermediate in non-survivors may indicate a disruption of sialic acid metabolism, potentially linked to downstream accumulation or altered turnover of N-acetylneuraminic acid, which has been previously associated with chronic kidney disease severity, end-stage renal disease, and adverse cardiovascular outcomes [45,46].

In contrast, indole-3-ethanol, also known as tryptophol, is a metabolite linked to tryptophan metabolism and microbial-derived pathways [47]. Enhanced levels of indole-3-ethanol have been associated with increased lymphocyte apoptosis in vitro, possibly mediated by the formation of DNA cross-links and aldehyde-protein adducts [48]. In this cohort, although not statistically significant, deceased patients showed decreased median lymphocyte counts. Since indole-3-ethanol levels showed a moderate association with age, an age-adjusted sensitivity analysis was performed. Although the association was attenuated after adjustment, indole-3-ethanol remained associated with mortality, suggesting that age-related metabolic variation may contribute to, but does not fully explain, the observed relationship (Table S9). Altered levels of this metabolite were also reported in microbial gut dysbiosis [49]. Notably, due to the untargeted nature of the analysis, the identification of indole-3-ethanol was not unequivocal, as the same m/z and retention time may correspond to alternative adducts, including nicotine-1′-N-oxide, highlighting potential ambiguity in metabolite annotation. Metabolite annotation was supported by accurate mass, retention time, and MS/MS fragmentation pattern analysis. Nevertheless, confirmation using authentic analytical standards, corresponding to the highest confidence level according to the Metabolomics Standards Initiative, is warranted in future targeted studies to establish the definitive identity of this feature and strengthen its biological interpretation.

Despite this, both candidate identities converge on biologically plausible pathways related to metabolic stress and inflammatory response [50]. These findings suggest that the observed metabolomic signature may capture underlying alterations in host metabolism and immune regulation associated with outcome. However, given the inherent limitations of untargeted metabolomics, further targeted validation is warranted to confirm metabolite identity and enable more precise biological interpretation.

The best-performing ECMO cohort multivariate model was composed of two molecules: α-carboxyethyl hydrochroman (α-CECH) and octanoate. Both metabolites retained their association with outcome throughout univariate and multivariate analysis. The model yielded good overall performance; however, cross-validation revealed some variability, highlighting limited stability across resampling iterations. α-CECH is a by-product of α-tocopherol metabolism, processed in the liver through cytochrome P450-mediated pathways [51]. Elevated levels of this molecule in non-survivors may reflect increased oxidative stress and altered lipid antioxidant metabolism, potentially associated with systemic inflammatory response and hepatic metabolic dysregulation in critical illness [52,53]. Consistent with this interpretation, α-CECH has previously been reported in association with sepsis severity in ICU populations [54].

Octanoate was also found at higher levels in ECMO non-survivors. This metabolite is a mitochondrial medium-chain fatty acid involved both as a substrate in β-oxidation and as a precursor in lipoic acid biosynthesis [55]. Its accumulation may reflect dysregulated mitochondrial fatty acid metabolism and systemic metabolic stress in critical illness [55]. Elevated octanoate levels have previously been associated with ARDS severity and renal dysfunction, supporting its potential role as a marker of multi-organ metabolic impairment [13,56].

**Figure 14:**
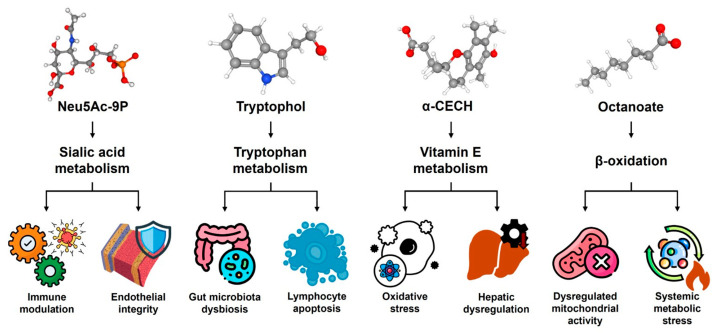
Putative biological interpretation of metabolomic signatures associated with clinical outcomes in IMV and ECMO cohorts. Illustrative representation of the biological context potentially associated with the candidate metabolites identified in the multivariate models. In the IMV cohort, N-acetyl-β-neuraminate 9-phosphate (Neu5Ac-9P) and indole-3-ethanol (tryptophol) were associated with sialic acid and tryptophan metabolism, respectively, whereas in the ECMO cohort, α-carboxyethyl hydrochroman (α-CECH) and octanoate were linked to vitamin E metabolism and mitochondrial fatty acid β-oxidation, respectively. The proposed biological connections illustrate potential mechanisms through which altered metabolite levels may reflect disturbances in immune regulation, oxidative stress, endothelial function, and mitochondrial metabolism during critical illness. These pathways represent hypothesis-generating interpretations based on current metabolic knowledge and require further targeted validation to establish metabolite identity, pathway involvement, and biological relevance.

An illustrative representation of the hypothesized biological implication of the candidate metabolites associated with the candidate metabolites identified in the multivariate models for the IMV and ECMO cohorts is presented in Figure 14.

Collectively, these metabolites converge on pathways involved in immune regulation, oxidative stress, mitochondrial energy metabolism, and endothelial dysfunction, suggesting that mortality in critically ill patients is characterized by a coordinated disruption of metabolic homeostasis rather than isolated alterations in individual metabolites.

### 4.3. Proteomics

The best-performing proteomic model in the IMV cohort included HLA-DQA1 and TRAV16, both of which consistently exhibited a stable protective association with ICU mortality across almost all cross-validation folds and in the full dataset. Biologically, these proteins are central to adaptive immune function, with HLA-DQA1 involved in MHC class II antigen presentation in antigen-presenting cells (e.g., B Lymphocytes, dendritic cells, macrophages) [57] and TRAV16 representing a component of the CD4^+^ T-cell receptor alpha repertoire.

In the case of HLA-DQA1, its lower abundance in non-survivors may reflect impaired antigen presentation capacity by professional antigen-presenting cells, rather than a direct reduction in lymphocyte counts. As a key component of the MHC class II complex, HLA-DQA1 is involved in antigen presentation leading to CD4+ T cell activation [58,59], and its downregulation may contribute to the immunosuppressive phenotype frequently observed in critically ill patients [60]. This mechanism is consistent with previous observations linking reduced CD4+ T-cell responses and immune dysfunction to increased disease severity in conditions such as COVID-19 and mortality in ARDS [61,62]. In the case of TRAV16, its lower abundance in non-survivors may reflect alterations in the T-cell receptor repertoire and reduced capacity for antigen recognition. As a variable segment of the T-cell receptor alpha chain, TRAV16 contributes to the diversity and specificity of antigen recognition by T lymphocytes [63]. Its reduced abundance may therefore reflect T-cell exhaustion and incapability to mount a proper adaptive immune response, which has been associated with worse outcomes in patients receiving invasive ventilatory support [64]. When considered alongside the decreased levels of HLA-DQA1, these findings suggest a coordinated disruption of both antigen presentation and T-cell recognition pathways, supporting the presence of an immunosuppressed phenotype associated with poor outcomes.

The ECMO cohort analysis resulted in a model composed of a single variable, IL-10. Although the model did not yield statistical significance across the majority of cross-validation folds, the variable itself remained significantly associated with the outcome in four folds as well as in the full model. This suggests that, despite limited predictive stability, IL-10 may capture an underlying biological signal related to the outcome. IL-10 is a key anti-inflammatory cytokine that limits excessive immune activation through inhibition of pro-inflammatory cytokine release and antigen presentation [65]. In the context of critical illness, IL-10 is often upregulated as part of a compensatory anti-inflammatory response aimed at controlling systemic inflammation [66]. In this study, higher IL-10 levels were associated with reduced mortality risk, which may reflect a more effective regulation of the inflammatory response and prevention of uncontrolled immune-mediated tissue damage.

Together, these proteins point towards impaired adaptive immunity, characterized by defective antigen presentation and reduced T-cell recognition capacity, supporting the concept of immunological exhaustion as a hallmark of poor outcome in critically ill patients.

### 4.4. Limitations

The ECMO cohort presented inherent challenges for multivariate modeling due to both biological and statistical factors. From a clinical perspective, ECMO-supported patients constitute a highly heterogeneous population, characterized by severe underlying illness, multiple competing mechanisms of mortality, and the additional physiological perturbations introduced by the extracorporeal circuit itself. These factors likely contribute to increased biological variability and reduced signal homogeneity, limiting the detectability of consistent omics signatures. This is further emphasized by the substantially smaller sample size in this cohort, resulting in constraints on model stability and increasing susceptibility to overfitting.

Relative to proteome complexity and proteoform diversity, the present proteomic workflow primarily evaluated differentially abundant protein-associated features at the peptide/protein annotation level and was not specifically designed to characterize distinct proteoforms arising from alternative splicing, post-translational modifications, or protein cleavage products. Consequently, the biological signals associated with proteins such as HLA-DQA1 and TRAV16 may reflect multiple underlying protein species and functional states that could not be fully resolved within the current untargeted approach. Given the profound proteomic remodeling observed during critical illness and systemic inflammation, future studies employing dedicated proteoform-resolved strategies may provide additional mechanistic insight into immune dysregulation associated with IMV and ECMO outcomes.

From a statistical standpoint, the combination of high-dimensional omics data with limited sample size led to issues of metabolites not passing through multiple corrections, model instability, including quasi-separation and wide confidence intervals across cross-validation folds. Although some models exhibited high apparent discrimination in the full dataset, this performance was not consistently reproduced during internal validation, indicating optimism in the full-model estimates. Additionally, feature selection was performed prior to cross-validation, which may introduce information leakage. Future studies with larger cohorts should incorporate nested cross-validation approaches, enabling feature selection and model optimization exclusively within training subsets.

No formal sample size estimate analysis was performed owing to the retrospective nature of the study and scarcity of eligible ECMO/IMV patients. Furthermore, the present findings represent exploratory and hypothesis-generating results, warranting cautious interpretation as further external validation in larger and independent cohorts is needed to confirm their robustness and generalizability.

## 5. Conclusions

Overall, the present study was able to identify potential biological signatures associated with ICU mortality across both IMV and ECMO cohorts using FTIRS, metabolomics, and proteomics approaches. Notably, the identified signatures were detectable several days before death, with a median time of sample collection to death of 9 (5–16) days in IMV and 15 (9–32) days in ECMO cohorts. These findings highlight the sensitivity of the applied omics platforms to capture clinically relevant biological alterations associated with critical illness progression and outcome. Additionally, the potential of integrated omics approaches to contribute toward precision medicine strategies was highlighted, improving prognostic assessment and guiding personalized interventions in critically ill patients.

## Figures and Tables

**Table 1:** Comparison of demographic, clinical, and laboratory characteristics between IMV survivors and non-survivors.

Variable	IMV Survivors (*n* = 15)	IMV Non-Survivors (*n* = 15)	*p*-Value *	Univariate Logistic Regression	
				Crude $\hat{OR}$ and 95% CI	*p*-Value
Demographics					
Age, years	55.00 (41.00–60.00)	60.00 (52.00–64.00)	**0.037**	1.126 (0.999–1.268)	0.052
Sex, male	7 (46.7%)	10 (66.7%)	0.462	0.438 (0.100–1.916)	0.273
BMI, kg/m^2^	27.34 (23.44–33.06)	29.22 (26.12–33.95)	0.305	1.048 (0.931–1.181)	0.437
Clinical data					
Arterial Hypertension	3 (20.0%)	9 (60.0%)	**0.025**	6.000 (1.172–30.725)	**0.032**
Diabetes Mellitus	2 (13.3%)	6 (40%9	0.215	4.333 (0.708–26.531)	0.113
Obesity	4 (26.7%)	9 (60.0%)	**0.065**	4.125 (0.883–19.273)	0.072
Dyslipidemia	3 (20.0%)	4 (26.7%)	0.999	1.455 (0.264–8.009)	0.667
Chronic respiratory disease	1 (6.67%)	5 (33.3%)	0.169	7.000 (0.705–69.490)	0.097
Laboratory data					
CRP, mg/mL	146.90 (73.00–262.50)	187.500 (99.00–281.60)	0.683	1.002 (0.994–1.10)	0.630
Lactate, mmol/L	1.40 (1.20–1.70)	1.70 (1.40–2.70)	**0.041**	3.438 (0.900–13.140)	0.071
Platelet count, minimum ×10^9^/L	292.00 (166.00–331.00)	219.00 (152.00–295.00)	0.412	0.998 (0.991–1.004)	0.447
Lymphocyte count, ×10^9^/L	1.40 (0.73–1.83)	0.830 (0.470–1.09)	0.089	0.928 (0.479–1.799)	0.825
Neutrophile count, ×10^9^/L	9.87 (6.81–11.44)	10.62 (7.09–15.13)	0.539	1.093 (0.925–1.292)	0.296
Leukocyte count, ×10^9^/L	12.60 (8.49–13.58)	12.14 (8.42–17.25)	0.902	1.047 (0.924–1.185)	0.471
Hemoglobin, ×10 g/L	11.30 (10.50–12.10)	10.30 (8.80–11.70)	0.217	0.834 (0.554–1.254)	0.383
INR	1.12 (1.08–1.13)	1.24 (1.07–1.35)	**0.041**	1.147 × 10^3^ (1.486–8.857 × 10^5^)	**0.038**
Bilirubin, mg/dL	0.63 (0.39–1.04)	0.560 (0.280–0.790)	0.624	1.077 (0.422–2.746)	0.877
Creatinine, mg/dL	0.69 (0.58–0.84)	0.810 (0.710–1.300)	0.098	1.792 (0.419–7.659)	0.431
Clinical intervention-related data					
ICU length of stay	18.00 (11.00–23.00)	14.00 (10.00–21.00)	0.217	0.912 (0.802–1.038)	0.165
ICU day at sample collection	6.00 (6.00–7.00)	6.00 (6.00–6.00)	0.217	0.001 (0.001–infinite)	0.999
Days on IMV	18.00 (8.00–20.00)	13.00 (9.00–20.00)	0.389	0.923 (0.812–1.050)	0.223
Days on IMV at sample collection	6.00 (6.00–7.00)	6.00 (5.00–6.00)	0.250	0.609 (0.287–1.293)	0.197
ICU length of stay from sample collection to outcome	13.00 (5.00–18.00)	9.00 (5.00–16.00)	0.345	0.926 (0.816–1.050)	0.231

Data are presented as median and interquartile range (P25–P75) for continuous variables, and as count (**n**) and percentage for categorical variables. * Mann–Whitney U *p-*values are reported for continuous variables; remaining *p*-values were obtained using the Chi-squared test or Fisher’s exact test, as appropriate. Significant *p-*values are highlighted in **bold**. Abbreviations: BMI—Body mass index; CRP—C-reactive protein; IMV—Invasive mechanical ventilation; INR—International normalized ratio.

**Table 2:** Comparison of demographic, clinical, and laboratory characteristics between ECMO survivors and non-survivors.

Variable	ECMO Survivors (*n* = 13)	ECMO Non-Survivors (*n* = 9)	*p*-Value *	Univariate Logistic Regression	
				Crude $\hat{OR}$ and 95% CI	*p*-Value
Demographics					
Age, years	46.00 (40.00–54.00)	58.00 (52.50–62.50)	**0.007**	1.131 (1.001–1.278)	**0.048**
Sex, male	6 (46.2%)	8 (88.9%)	0.074	0.107 (0.010–1.121)	0.107
BMI, kg/m^2^	32.653 (27.513–35.101)	29.218 (26.187–33.798)	0.647	1.000 (0.873–1.144)	0.995
Clinical data					
Arterial Hypertension	3 (23.1%)	4 (44.4%)	0.376	2.667 (0.423–16.826)	0.297
Diabetes Mellitus	1 (7.7%)	3 (33.3%)	0.264	6.000 (0.509–70.668)	0.154
Obesity	9 (69.2%)	4 (44.4%)	0.235	0.356 (0.061–2.077)	0.251
Dyslipidemia	0 (0.0%)	3 (33.3%)	-	3.5001 × 10^9^ (0.001–infinite)	0.999
Chronic respiratory disease	1 (7.7%)	2 (22.2%)	0.544	3.429 (0.261–45.026)	0.348
Laboratory data					
CRP, mg/mL	214.70 (87.45–267.80)	232.40 (115.90–308.55)	0.896	0.999 (0.993–1.005)	0.770
Lactate, mmol/L	1.50 (1.00–1.70)	2.00 (1.30–2.45)	0.292	2.056 (0.585–7.227)	0.261
Platelet count, ×10^9^/L	196.00 (153.50–299.50)	157.00 (119.00–237.00)	0.235	0.993 (0.983–1.004)	0.204
Lymphocyte count, ×10^9^/L	1.02 (0.66–2.15)	1.20 (0.92–1.58)	0.556	0.799 (0.357–1.788)	0.585
Neutrophile count, ×10^9^/L	15.88 (13.33–19.19)	11.80 (8.00–14.74)	0.060	0.784 (0.609–1.010)	0.060
Leukocyte count, ×10^9^/L	17.60 (14.61–22.51)	14.13 (10.21–18.23)	0.071	0.836 (0.678–1.031)	0.095
Hemoglobin, ×10 g/L	9.10 (8.75–10.80)	8.90 (7.85–10.05)	0.324	0.759 (0.403–1.428)	0.392
INR	1.12 (1.02–1.25)	1.17 (1.08–1.25)	0.512	3.447 (0.091–131.146)	0.505
Bilirubin, mg/dL	0.58 (0.51–0.97)	1.07 (0.47–1.85)	0.186	4.776 (0.558–40.851)	0.153
Creatinine, mg/dL	0.76 (0.51–0.89)	0.85 (0.6–1.11)	0.262	3.118 (0.274–35.528)	0.360
Clinical intervention-related data					
ICU length of stay	30.00 (22.00–64.00)	22.00 (17.00–37.50)	0.110	0.956 (0.906–1.009)	0.105
ICU day at sample collection	6.00 (6.00–7.00)	7.00 (6.00–10.00)	0.431	1.286 (0.814–2.034)	0.281
Days on IMV	43.00 (20.50–60.50)	21.00 (17.50–40.50)	0.164	0.963 (0.915–1.014)	0.150
Days on IMV at sample collection	6.00 (6.00–7.00)	8.00 (6.50–12.50)	**0.030**	1.538 (1.003–2.356)	**0.048**
Days on ECMO	22.00 (11.00–47.00)	19.00 (11.50–31.00)	0.695	0.980 (0.926–1.036)	0.472
Days on ECMO at sample collection	6.00 (1.00–6.50)	6.00 (6.00–6.50)	0.512	1.257 (0.820–1.926)	0.293
ICU length of stay from sample collection to outcome	23 (16.5–58.5)	15.00 (9.00–32.00)	0.051	0.953 (0.902–1.007)	0.088

Data are presented as median and interquartile range (P25–P75) for continuous variables, and as count (*n*) and percentage for categorical variables. * Mann–Whitney U *p-*values are reported for continuous variables; remaining *p*-values were obtained using the Chi-squared test or Fisher’s exact test, as appropriate. Significant *p-*values are highlighted in bold. Abbreviations: BMI—Body mass index; CRP—C-reactive protein; IMV—Invasive mechanical ventilation; INR—International normalized ratio; ECMO—Extracorporeal membrane oxygenation.

**Table 3:** FTIRS adjusted models on the IMV and ECMO cohorts.

Adjusted Models	Multivariable Models				Model Performance		
	$a\hat{OR}$	95% CI	*p*-Value	AIC	AUC	95% CI	*p-*Value
IMV Cohort—ATM + BC							
1365 cm^−1^	N.E						
1364 cm^−1^							
1363 cm^−1^							
IMV cohort—ATM + BC + UVN							
1587 cm^−1^	N.E						
1357 cm^−1^							
1343 cm^−1^							
IMV cohort—ATM + SGolay							
1529 cm^−1^	0.019	0.001–3.715	0.141	25.272	0.982	0.933–1.00	<0.001
837 cm^−1^	0.019	0.001–11.833	0.228				
744 cm^−1^	0.011	0.001–6.952	0.171				
ECMO cohort—ATM + SGolay							
601 cm^−1^	1528.285	0.119–1.962 × 10^7^	0.129	22.621	0.948	0.841–1.00	<0.001
770 cm^−1^	3.871 × 10^−6^	0.001–14.589	0.107				

Adjusted odds-ratio ($a\hat{OR}$) and their corresponding 95% confidence intervals were estimated using the full dataset. IMV cohort models were adjusted for the following variables: age, presence of arterial hypertension, lactate, and INR. ECMO cohort models were adjusted for the following variables: age and days on IMV at sample collection. Abbreviations: ATM + BC: Atmospheric + Baseline corrections; ATM + BC + UVN: Atmospheric + Baseline corrections and Unit Vector Normalization; ATM + SGolay: Atmospheric correction and Savitzky–Golay derivative; $a\hat{OR}$: Adjusted odds-ratio; 95% CI: 95% Confidence Interval; AIC: Akaike Information Criterion; AUC: area under the curve; N.E.: not estimable due to model non-convergence, quasi-separation, or unstable coefficient estimation.

**Table 4:** Metabolomics and Proteomics adjusted models for IMV and ECMO cohorts.

Adjusted Models	Multivariable Models				Model Performance		
	$a\hat{OR}$	95% CI	*p-*Value	AIC	AUC	95% CI	*p-*Value
IMV Cohort—Metabolomics							
N-acetyl-β-neuraminate 9-phosphate	8.036 × 10^−217^	N.E.	0.999	14.205	1.00	1.00–1.00	<0.001
indole-3-ethanol	N.E.		0.999				
ECMO cohort—Metabolomics							
α-carboxyethyl-hydroxychroman	N.E			N.E			
Octanoate							
IMV cohort—Proteomics							
TRAV 16	0.864	0.697–1.072	0.186	35.656	0.916	0.808–1.00	<0.001
HLA-DQA1	0.761	0.573–1.013	0.061				
ECMO cohort—Proteomics							
IL10	0.001	0.001–2.577	0.087	22.00	0.974	0.898–1.00	<0.001

Adjusted odds-ratio ($a\hat{OR}$) and their corresponding 95% confidence intervals were estimated using the full dataset. IMV cohort models were adjusted for the following variables: age, presence of arterial hypertension, lactate, and INR. ECMO cohort models were adjusted for the following variables: age and days on IMV at sample collection. Abbreviations: $a\hat{OR}$: Adjusted odds-ratio; 95% CI: 95% Confidence-Interval; AIC: Akaike Information Criterion; AUC: area under the curve; N.E.: not estimable due to model non-convergence, quasi-separation, or unstable coefficient estimation.

## Data Availability

The datasets generated and analyzed during the current study are openly available through the Zenodo repository. Due to file size limitations, some datasets were deposited in multiple parts. The available datasets include proteomics and metabolomics raw data for ECMO and non-ECMO patients stratified according to outcome (deceased or discharged). https://doi.org/10.5281/zenodo.20257823; https://doi.org/10.5281/zenodo.20258117; https://doi.org/10.5281/zenodo.20258329; https://doi.org/10.5281/zenodo.20258495; https://doi.org/10.5281/zenodo.20259141; https://doi.org/10.5281/zenodo.20259288; https://doi.org/10.5281/zenodo.20259814; https://doi.org/10.5281/zenodo.20259943; https://doi.org/10.5281/zenodo.20260420; https://doi.org/10.5281/zenodo.20260812; https://doi.org/10.5281/zenodo.20261414; https://doi.org/10.5281/zenodo.20261665; https://doi.org/10.5281/zenodo.20261774; https://doi.org/10.5281/zenodo.20261908; https://doi.org/10.5281/zenodo.20262027; https://doi.org/10.5281/zenodo.20262120; https://doi.org/10.5281/zenodo.20262233; https://doi.org/10.5281/zenodo.20264512; https://doi.org/10.5281/zenodo.20264786; https://doi.org/10.5281/zenodo.20264934; https://doi.org/10.5281/zenodo.20265162.

## Supplementary Materials

The following supporting information can be downloaded at: https://www.mdpi.com/article/10.3390/metabo16070516/s1. Figure S1. Comparison of disease severity indices between IMV survivors and non-survivors. Figure S2. Comparison of disease severity indices between ECMO survivors (orange) and non-survivors. Figure S3. Correlation between metabolite intensities and age. Table S1. Multivariate logistic regression models derived from different FTIR preprocessing approaches (ATM+BC, ATM+BC+UVN, and Savitzky-Golay) and their predictive performance metrics, for mortality prediction on IMV patients. Table S2. Multivariate logistic regression models derived from different FTIR preprocessing approaches (Savitzky-Golay) and their predictive performance metrics, for mortality prediction on ECMO patients. Table S3. Best-performing multivariate regression model derived from metabolomic data for mortality prediction in IMV patients. Table S4. Best-performing multivariate regression model derived from metabolomic data for mortality prediction in ECMO patients. Table S5. Multivariate logistic regression model for IMV mortality prediction based on proteomic data. Table S6. Multivariate logistic regression model for ECMO mortality prediction based on proteomic data. Table S7. FTIRS Adjusted models on the IMV and ECMO cohorts. Table S8. Metabolomics and Proteomics adjusted models for IMV and ECMO cohorts. Table S9. Effect of age adjustment on the association between indole-3-ethanol and mortality outcome on the IMV cohort. Table S10. Multivariable regression models based on demographic and clinical variables for mortality prediction in IMV and ECMO cohorts.
