# Supplementary material for: Metabolomic Signatures of Biotrauma Associated with Mortality in ICU Patients Requiring Invasive Mechanical Ventilation and ECMO
Source: Metabolites. 2026 Jul 22;16(7):516. doi: 10.3390/metabo16070516 (PMC13414384; doi:10.3390/metabo16070516)
Supplement: Supplementary file 1 [file metabolites-16-00516-s001.zip › metabolites-4429486-supplementary.pdf]

Metabolomic Signatures of Biotrauma Associated with Mortality in ICU Patients Requiring Invasive Mechanical Ventilation and ECMO

Tiago A. H. Fonseca [1,2,3], Cristiana P. Von Rekowski [1,2,3], Rúben Araújo [1,2,3], Gonçalo C. Justino [4], M. Conceição Oliveira [4], Luís Bento [5,6,7] and Cecília R. C. Calado [2,8,*]

1 NMS—NOVA Medical School, FCM—Faculdade de Ciências Médicas, Universidade NOVA de Lisboa, Campo dos Mártires da Pátria 130, 1169-056 Lisbon, Portugal; tiago.alexandre.hf@gmail.com (T.A.H.F.); cristiana.vonrekowski@gmail.com (C.P.V.R.); rubenalexandredinisaraujo@gmail.com (R.A.)
2 ISEL—Instituto Superior de Engenharia de Lisboa, Instituto Politécnico de Lisboa, Rua Conselheiro Emídio Navarro 1, 1959-007 Lisbon, Portugal
3 CHRC—Comprehensive Health Research Centre, Universidade NOVA de Lisboa, 1150-082 Lisbon, Portugal
4 Centro de Química Estrutural—Institute of Molecular Sciences, Instituto Superior Técnico, Universidade de Lisboa, Av. Rovisco Pais 1, 1049-001 Lisbon, Portugal; goncalo.justino@tecnico.ulisboa.pt (G.C.J.); conceicao.oliveira@tecnico.ulisboa.pt (M.C.O.)
5 CHRC—Comprehensive Health Research Centre, NMS—NOVA Medical School, FCM—Faculdade de Ciências Médicas, Universidade NOVA de Lisboa, 1169-056 Lisbon, Portugal; luis.bento@ulssjose.min-saude.pt
6 Intensive Care Department, ULS São José—Unidade Local de Saúde São José, Rua José António Serrano, 1150-199 Lisbon, Portugal;
7 CCAL—Centro Clínico Académico de Lisboa, 1649-028 Lisbon, Portugal
8 iBB—Institute for Bioengineering and Biosciences, i4HB—The Associate Laboratory Institute for Health and Bioeconomy, IST—Instituto Superior Técnico, Universidade de Lisboa, Av. Rovisco Pais, 1049-001 Lisbon, Portugal
* Correspondence: cecilia.calado@isel.pt

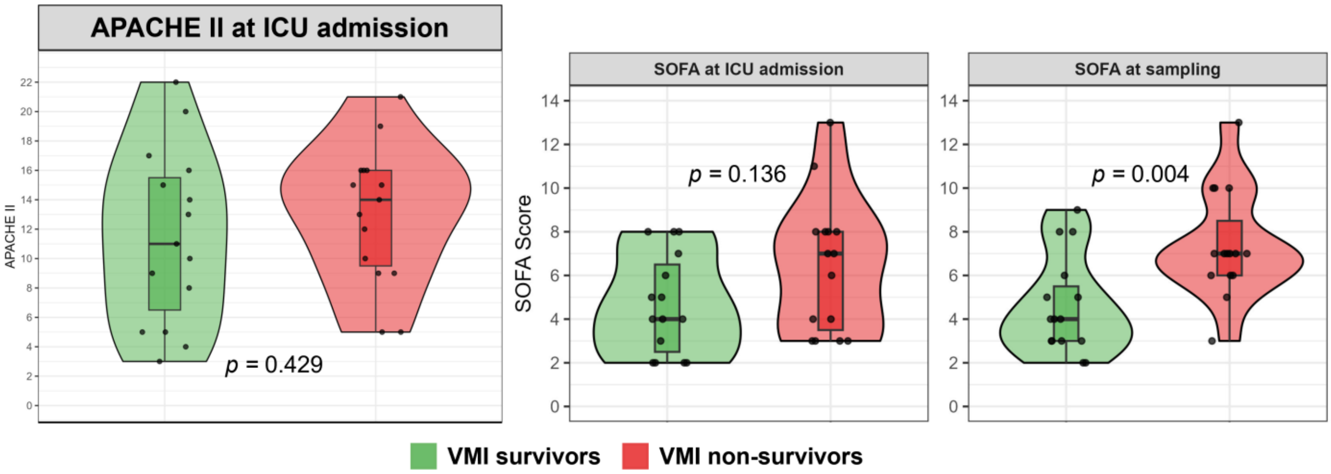

Figure S1 - Comparison of disease severity indices between IMV survivors (green) and non-survivors (red). APACHE II score at ICU admission, SOFA score at ICU admission, and SOFA score at the time of serum sampling are shown. Violin plots represent the distribution (density) of the data, while boxplots indicate the median (horizontal line), interquartile range (box), and whiskers extending to 1.5 × the interquartile range. Individual patients are represented by black dots. Statistical comparisons between groups were performed using the Mann–Whitney U test, with p-values displayed within each panel.

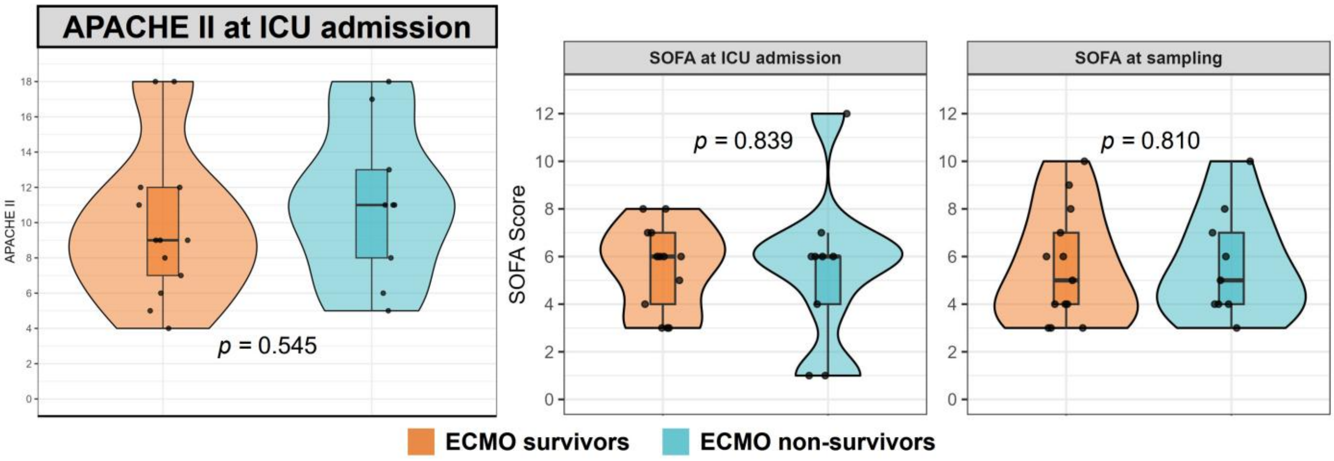

Figure S2 - Comparison of disease severity indices between ECMO survivors (orange) and non-survivors (blue). APACHE II score at ICU admission, SOFA score at ICU admission, and SOFA score at the time of serum sampling are shown. Violin plots represent the distribution (density) of the data, while boxplots indicate the median (horizontal line), interquartile range (box), and whiskers extending to 1.5 × the interquartile range. Individual patients are represented by black dots. Statistical comparisons between groups were performed using the Mann–Whitney U test, with p-values displayed within each panel

Table S1 - Multivariate logistic regression models derived from different FTIR preprocessing approaches (ATM+BC, ATM+BC+UVN, and Savitzky-Golay) and their predictive performance metrics, for mortality prediction on IMV patients.

**FTIRs Models**	**Multivariable Models**				**Model Performance**			**Discrimination**		
	$a\widehat{OR}$	**95% CI**	**p–value**	**AIC***	**AUC***	**95% CI***	**p–value***	**ACC (95% CI)**	**Sen (95% CI)**	**Spe (95% CI)**
ATM+BC										
1365 cm^{-1}	229.196	1.165 - 4.51 x10^{4}	0.0437	19.872 ± 3.462	0.956 ± 0.054	0.849-1.00	0.004	0.933 (0.787 - 0.982)	0.933 (0.702 - 0.988)	0.933 (0.702 - 0.988)
1364 cm^{-1}	1.279 x10^{-6}	8.058 x10^{-12} - 0.203	0.0263							
1363 cm^{-1}	3754.155	3.966 - 3.553 x10^{6}	0.0185							
ATM+BC+UVN										
1587 cm^{-1}	1.428	1.001 - 2.037	0.0489	16.316 ± 4.448	0.933 ± 0.0889	0.759-1.00	0.038	0.933 (0.787-0.982)	0.933 (0.702 - 0.988)	0.933 (0.702 - 0.988)
1357 cm^{-1}	46.27	1.494 - 1433.219	0.0286							
1343 cm^{-1}	0.015	3.419 x10^{-4} - 0.671	0.0303							
ATM+SGolay										
1529 cm^{-1}	0.040	0.002 - 0.798	0.035	14.182 ± 3.362	0.911 ± 0.083	0.748-1.074	0.041	0.933 (0.787 - 0.982)	0.933 (0.702 - 0.988)	0.933 (0.702 - 0.988)
837 cm^{-1}	0.058	0.004 - 0.860	0.038							
744 cm^{-1}	0.036	0.001 - 0.975	0.048							

Adjusted odds-ratio ($a\widehat{OR}$) and their corresponding 95% confidence were estimated using the full dataset. *Performance metrics are presented as mean ± standard deviations and were estimated using 5-fold stratified cross-validation (AIC, AUC, AUC 95% confidence interval, AUC p-value). Discrimination metrics are presented with the values of the full dataset accompanied by the 95% confidence interval. (**Abbreviations**: ATM+BC: Atmospheric + Baseline corrections; ATM+BC+UVN: Atmospheric + Baseline corrections and Unit Vector Normalization; ATM+SGolay: Atmospheric correction and Savitzky-Golay derivative; $a\widehat{OR}$: Adjusted odds-ratio; 95% CI: 95% Confidence-Interval; AIC: Akaike Information Criterion; AUC: area under the curve; ACC: Accuracy; Sen: sensibility; Spe: Specificity).

Table S2 - Multivariate logistic regression models derived from different FTIR preprocessing approaches (Savitzky-Golay) and their predictive performance metrics, for mortality prediction on ECMO patients.

**FTIRs Models**	**Multivariable Models**				**Model Performance**			**Discrimination**		
	$a\widehat{OR}$	**95% CI**	**p–value**	**AIC***	**AUC***	**95% CI***	**p–value***	**ACC (95% CI)**	**Sen (95% CI)**	**Spe (95% CI)**
ATM+SGolay										
601,728	244.995	1.357 - 4.422 x10^{4}	0.038	18.756 ± 3.370	0.967 ± 0.067	0.836-1.00	0.0252 ± 0.050	0.863 (0.667-0.953)	0.777 (0.453-0.937)	0.923 (0.667-0.986)
770,482	3.547 x10^{-5}	2.411 x10^{-9} - 0.522	0.036							

Adjusted odds-ratio ($a\widehat{OR}$) and their corresponding 95% confidence were estimated using the full dataset. *Performance metrics (AIC and AUC) are presented as mean ± standard deviations and were estimated using 5-fold stratified cross-validation (AIC, AUC, AUC 95% confidence interval, AUC p-value). Discrimination metrics are presented with the values of the full dataset accompanied by the 95% confidence interval. (**Abbreviations**: ATM+BC: Atmospheric + Baseline corrections; ATM+BC+UVN: Atmospheric + Baseline corrections and Unit Vector Normalization; ATM+SGolay: Atmospheric correction and Savitzky-Golay derivative; $a\widehat{OR}$: Adjusted odds-ratio; 95% CI: 95% Confidence-Interval; AIC: Akaike Information Criterion; AUC: area under the curve; ACC: Accuracy; Sen: sensibility; Spe: Specificity).

Table S3 - Best-performing multivariate regression model derived from metabolomic data for mortality prediction in IMV patients.

Metabolomics	Multivariable Models				Model Performance			Discrimination		
	$a\widehat{OR}$	95% CI	p–value	AIC*	AUC*	95% CI*	p–value*	Acc (95% CI)	Sen (95% CI)	Spe (95% CI)
N-acetyl-β-neuraminate 9-phosphate	0.057	0.005 - 0.599	0.017	13.82 ± 3.991	0.944 ± 0.070	0.807-1.00	0.0162 ± 0.027	0.900 (0.744-0.965)	0.933 (0.702-0.988)	0.867 (0.621-0.963)
indole-3-ethanol	178.061	1.492 - 2.124 x10^4	0.034							

Adjusted odds-ratio ($a\widehat{OR}$) and their corresponding 95% confidence were estimated using the full dataset. *Performance metrics (AIC and AUC) are presented as mean ± standard deviations and were estimated using 5-fold stratified cross-validation (AIC, AUC, AUC 95% confidence interval, AUC p-value). Discrimination metrics are presented with the values of the full dataset accompanied by the 95% confidence interval. (**Abbreviations**: $a\widehat{OR}$: Adjusted odds-ratio; 95% CI: 95% Confidence-Interval; AIC: Akaike Information Criterion; AUC: area under the curve; AUC: area under the curve; Acc: Accuracy; Sen: sensibility; Spe: Specificity).

Table S4 - Best-performing multivariate regression model derived from metabolomic data for mortality prediction in ECMO patients.

Metabolomics	Multivariable Models				Model Performance			Discrimination		
	$a\widehat{OR}$	95% CI	p–value	AIC*	AUC*	95% CI*	p–value*	Acc (95% CI)	Sen (95% CI)	Spe (95% CI)
α-carboxy-ethylhydroxychroman	6.262	1.123 - 34.917	0.036	17.210 ± 3.282	0.867 ± 0.194	0.486-1.00	0.225 ± 0.390	0.863 (0.667-0.953)	0.777 (0.453-0.937)	0.923 (0.667-0.986)
Octanoate	16.401	1.125 - 238.961	0.041							

Adjusted odds-ratio ($a\widehat{OR}$) and their corresponding 95% confidence were estimated using the full dataset. *Performance metrics (AIC and AUC) are presented as mean ± standard deviations and were estimated using 5-fold stratified cross-validation (AIC, AUC, AUC 95% confidence interval, AUC p-value). Discrimination metrics are presented with the values of the full dataset accompanied by the 95% confidence interval. (**Abbreviations**: $a\widehat{OR}$: Adjusted odds-ratio; 95% CI: 95% Confidence-Interval; AIC: Akaike Information Criterion; AUC: area under the curve; Acc: Accuracy; Sen: sensibility; Spe: Specificity).

Table S5 - Multivariate logistic regression model for IMV mortality prediction based on proteomic data.

Proteomics	Multivariable Models				Model Performance			Discrimination		
	$a\widehat{OR}$	95% CI	p–value	AIC*	AUC*	95% CI*	p–value*	Acc (95% CI)	Sen (95% CI)	Spe (95% CI)
TRAV16_79B	0.841	0.712 - 0.992	0.041	27.732 ± 1.340	0.889 ± 0.070	0.751 - 1.00	0.0427 ± 0.069	0.767 (0.591-0.882)	0.867 (0.621-0.963)	0.667 (0.417-0.848)
HLA-DQA1_79B	0.732	0.568 - 0.943	0.016							

Adjusted odds-ratio ($a\widehat{OR}$) and their corresponding 95% confidence were estimated using the full dataset. *Performance metrics (AIC and AUC) are presented as mean ± standard deviations and were estimated using 5-fold stratified cross-validation (AIC, AUC, AUC 95% confidence interval, AUC p-value). Discrimination metrics are presented with the values of the full dataset accompanied by the 95% confidence interval. (**Abbreviations**: TRAV 16: T cell receptor alpha variable 16; HLA-DQA1: MHC class II antigen DQA1; $a\widehat{OR}$: Adjusted odds-ratio; 95% CI: 95% Confidence-Interval; AIC: Akaike Information Criterion; AUC: area under the curve; Acc: Accuracy; Sen: sensibility; Spe: Specificity).

Table S6 - Multivariate logistic regression model for ECMO mortality prediction based on proteomic data.

Proteomics	Multivariable Models				Model Performance			Discrimination		
	$a\widehat{OR}$	95% CI	p–value	AIC*	AUC*	95% CI*	p–value*	Acc (95% CI)	Sen (95% CI)	Spe (95% CI)
IL-10	0.049	0.004 - 0.673	0.024	20.260 ± 1.750	0.783 ± 0.194	0.402-1.00	0.402 ± 0.385	0.727 (0.518-0.868)	0.667 (0.354-0.879)	0.769 (0.497-0.918)

Adjusted odds-ratio ($a\widehat{OR}$) and their corresponding 95% confidence were estimated using the full dataset. *Performance metrics (AIC and AUC) are presented as mean ± standard deviations and were estimated using 5-fold stratified cross-validation (AIC, AUC, AUC 95% confidence interval, AUC p-value). Discrimination metrics are presented with the values of the full dataset accompanied by the 95% confidence interval. (**Abbreviations**: IL-10: Interleukin-10; $a\widehat{OR}$: Adjusted odds-ratio; 95% CI: 95% Confidence-Interval; AIC: Akaike Information Criterion; AUC: area under the curve; Acc: Accuracy; Sen: sensibility; Spe: Specificity).

Table S7 - FTIRS Adjusted models on the IMV and ECMO cohorts.

Adjusted Models	Multivariable Models				Model Performance			Discrimination		
	$a\widehat{OR}$	95% CI	p–value	AIC	AUC	95% CI	p–value	Acc (95% CI)	Sen (95% CI)	Spe (95% CI)
**IMV Cohort - *ATM+BC***										
1365 cm^{-1}	N.E									
1364 cm^{-1}										
1363 cm^{-1}										
**IMV cohort - *ATM+BC+UVN***										
1587 cm^{-1}	N.E									
1357 cm^{-1}										
1343 cm^{-1}										
**IMV cohort - *ATM+SGolay***										
1529 cm^{-1}	0.019	0.001 - 3.715	0.141	25.272	0.982	0.933-1.00	<0.001	0.933 (0.787-0.982)	0.933 (0.702-0.988)	0.933 (0.702-0.988)
837 cm^{-1}	0.019	0.001 - 11.833	0.228							
744 cm^{-1}	0.011	0.001 - 6.952	0.171							
**ECMO cohort - *ATM+SGolay***										
601 cm^{-1}	1528.285	0.119 - 1.962 x10^{7}	0.129	22.621	0.948	0.841-1.00	<0.001	0.864 (0.667-0.953)	0.778 (0.453-0.937)	0.923 (0.667-0.986)
770 cm^{-1}	3.871 x10^{-6}	0.001 - 14.589	0.107							

Adjusted odds-ratio ($a\widehat{OR}$) and their corresponding 95% confidence were estimated using the full dataset. **IMV cohort models were adjusted for the following variables**: age, presence of arterial hypertension, lactate, and INR. **ECMO cohort models were adjusted for the following variables:** age, and days on IMV at sample collection. Discrimination metrics are presented with the values of the full dataset accompanied by the 95% confidence interval**. Abbreviations**: ATM+BC: Atmospheric + Baseline corrections; ATM+BC+UVN: Atmospheric + Baseline corrections and Unit Vector Normalization; ATM+SGolay: Atmospheric correction and Savitzky-Golay derivative; $a\widehat{OR}$: Adjusted odds-ratio; 95% CI: 95% Confidence-Interval; AIC: Akaike Information Criterion; AUC: area under the curve; N.E.: not estimable due to model non-convergence, quasi-separation, or unstable coefficient estimation; Acc: Accuracy; Sen: sensibility; Spe: Specificity.

Table S8 - Metabolomics and Proteomics adjusted models for IMV and ECMO cohorts.

Adjusted Models	Multivariable Models				Model Performance			Discrimination		
	$a\widehat{OR}$	95% CI	p–value	AIC	AUC	95% CI	p–value	Acc (95% CI)	Sen (95% CI)	Spe (95% CI)
**IMV Cohort - *Metabolomics***										
N-acetyl-β-neuraminate 9-phosphate	8.036 x10^{-217}	N.E.	0.999	14.205	1.00	1.00 - 1.00	<0.001	0.999 (0.886-1.000)	0.999 (0.796-1.000)	0.999 (0.796-1.000)
indole-3-ethanol	N.E.		0.999							
**ECMO cohort - *Metabolomics***										
α-carboxyethyl-hydroxychroman	N.E									
Octanoate										
**IMV cohort - *Proteomics***										
TRAV 16	0.864	0.697 - 1.072	0.186	35.656	0.916	0.808-1.00	<0.001	0.767 (0.591-0.882)	0.867 (0.621-0.963)	0.667 (0.417-0.848)
HLA-DQA1	0.761	0.573 - 1.013	0.061							
**ECMO cohort - *Proteomics***										
IL10	0.001	0.001 - 2.577	0.087	22.00	0.974	0.898-1.00	<0.001	0.909 (0.722-0.975)	0.889 (0.565-0.980)	0.923 (0.667-0.986)

Adjusted odds-ratio ($a\widehat{OR}$) and their corresponding 95% confidence were estimated using the full dataset. **IMV cohort models were adjusted for the following variables**: age, presence of arterial hypertension, lactate, and INR. **ECMO cohort models were adjusted for the following variables:** age, and days on IMV at sample collection. **Abbreviations**: $a\widehat{OR}$: Adjusted odds-ratio; 95% CI: 95% Confidence-Interval; AIC: Akaike Information Criterion; AUC: area under the curve; N.E.: not estimable due to model non-convergence, quasi-separation, or unstable coefficient estimation; Acc: Accuracy; Sen: sensibility; Spe: Specificity.

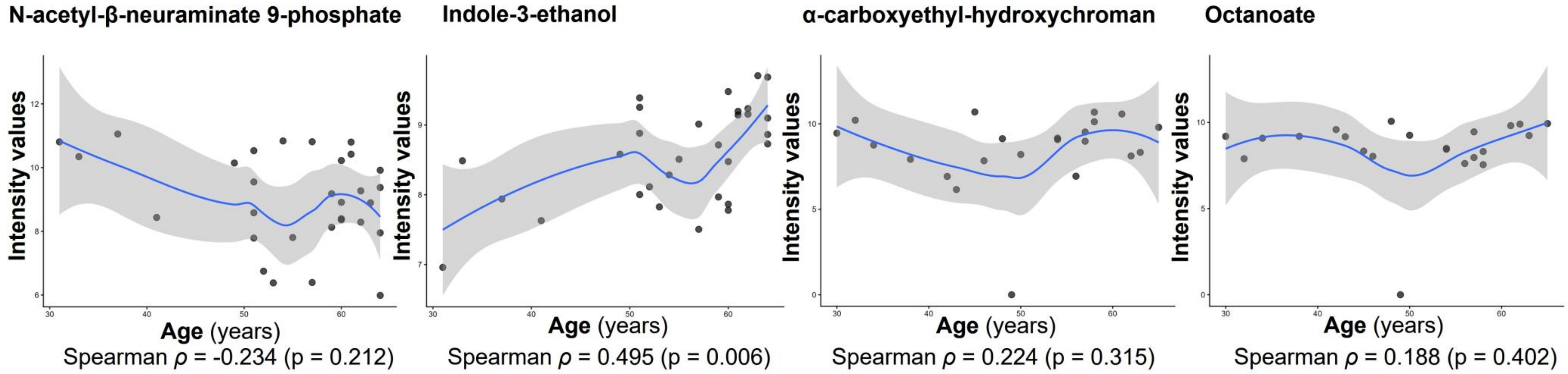

Figure S3 - Correlation between metabolite intensities and age. Spearman correlation analysis of selected metabolites showing their association with patient age. Each point represents an individual sample, the blue line indicates the LOESS fitted trend, and the shaded region represents the 95% confidence interval. Spearman's correlation coefficient (ρ) and p-values are shown for each metabolite. Indole-3-ethanol displayed a significant positive correlation with age, while the remaining metabolites showed no significant associations.

Table S9 - **Effect of age adjustment on the association between indole-3-ethanol and mortality outcome on the IMV cohort**. Unadjusted and age-adjusted logistic regression models were used to evaluate the association between indole-3-ethanol levels and mortality. Odds ratios (ORs), adjusted odds ratios (aORs), 95% confidence intervals (CIs), and p-values are reported.

Model	OR (95% CI)	p-value
Indole-3-ethanol (unadjusted)	6.791 (1.560 - 29.577)	0.011
Indole-3-ethanol + Age	4.922 (1.034-23.443)	0.045

Table S10 - Multivariable regression models based on demographic and clinical variables for mortality prediction in IMV and ECMO cohorts.

Adjusted Models	Multivariable Models			Model Performance				Discrimination		
	$a\widehat{OR}$	95% CI	p–value	AIC*	AUC*	95% CI*	p–value*	Acc (95% CI)	Sen (95% CI)	Spe (95% CI)
**IMV Cohort - *Demographics***										
Age (years)	1.074	0.926-1.247	0.344	31.598 ±1.503	0.778 ±0.070	0.271 - 0.934	0.207 ±0.153	0.800 (0.627-0.905)	0.800 (0.548-0.930)	0.800 (0.548-0.930)
Arterial Hypertension	5.389	0.680-42.727	0.108							
Lactate	1.290	0.274-6.076	0.747							
INR	1189.622	0.768-1.844 x10^6	0.059							
**ECMO cohort - *Demographics***										
Age (years)	1.11	0.983-1.246	0.094	21.791 ±1.778	0.833 ±0.211	0.599 - 1.00	0.309 ±0.40	0.772 (0.566-0.899)	0.667 (0.354-0.879)	0.846 (0.578-0.957)
Days IMV when sample was collected	1.513	0.924-2.476	0.095							

Adjusted odds-ratio ($a\widehat{OR}$) and their corresponding 95% confidence were estimated using the full dataset. *Performance metrics (AIC and AUC) are presented as mean ± standard deviations and were estimated using 5-fold stratified cross-validation (AIC, AUC, AUC 95% confidence interval, AUC p-value). Discrimination metrics are presented with the values of the full dataset accompanied by the 95% confidence interval. (**Abbreviations**: INR: International Normalized Ratio; $a\widehat{OR}$: Adjusted odds-ratio; 95% CI: 95% Confidence-Interval; AIC: Akaike Information Criterion; AUC: area under the curve; Acc: Accuracy; Sen: sensibility; Spe: Specificity).
